# Increased regulatory activity of intestinal innate lymphoid cells type 3 (ILC3) prevents experimental autoimmune encephalomyelitis severity

**DOI:** 10.1186/s12974-024-03017-7

**Published:** 2024-01-18

**Authors:** Milica Lazarević, Goran Stegnjaić, Bojan Jevtić, Sanja Despotović, Đurđica Ignjatović, Suzana Stanisavljević, Neda Nikolovski, Miljana Momčilović, Graeme L. Fraser, Mirjana Dimitrijević, Đorđe Miljković

**Affiliations:** 1 Department of Immunology, Institute for Biological Research “Siniša Stanković” - National Institute of Republic of Serbia, University of Belgrade, Despota Stefana 142, 11000 Belgrade, Serbia; 2 Institute of Histology and Embryology, School of Medicine, University of Belgrade, Dr Subotića 9, 11000 Belgrade, Serbia; 3 Department of Biochemistry, Institute for Biological Research “Siniša Stanković” - National Institute of Republic of Serbia, University of Belgrade, Despota Stefana 142, 11000 Belgrade, Serbia; 4 Epics Therapeutics S.A, 47 Rue Adrienne Bolland, 6041 Gosselies, Belgium

**Keywords:** Experimental autoimmune encephalomyelitis, Multiple sclerosis, Intestine, T cells, Innate lymphoid cells, Free fatty acid receptor type 2

## Abstract

Experimental autoimmune encephalomyelitis (EAE) induced in inbred rodents, i.e., genetically identical animals kept under identical environmental conditions, shows variable clinical outcomes. We investigated such variations of EAE in Dark Agouti rats immunized with spinal cord homogenate and identified four groups: lethal, severe, moderate, and mild, at day 28 post immunization. Higher numbers of CD4^+^ T cells, helper T cells type 1 (Th1) and 17 (Th17) in particular, were detected in the spinal cord of the severe group in comparison with the moderate group. In addition, increased proportion of Th1 and Th17 cells, and heightened levels of interferon (IFN)-γ and interleukin (IL)-6 were detected in the small intestine lamina propria of the severe group. A selective agonist of free fatty acid receptor type 2 (Ffar2) applied orally in the inductive phase of EAE shifted the distribution of the disease outcomes towards milder forms. This effect was paralleled with potentiation of intestinal innate lymphoid cells type 3 (ILC3) regulatory properties, and diminished Th1 and Th17 cell response in the lymph nodes draining the site of immunization. Our results suggest that different clinical outcomes in DA rats are under determinative influence of intestinal ILC3 activity during the inductive phase of EAE.

**Supplementary Information:**

The online version contains supplementary material available at 10.1186/s12974-024-03017-7.

## Introduction

Multiple sclerosis is an inflammatory, demyelinating, and neurodegenerative disease of the central nervous system (CNS) [1]. Experimental autoimmune encephalomyelitis (EAE) is a valuable model for exploring the pathogenesis of multiple sclerosis and evaluating the response to potential therapeutic approaches [2–4]. Immunization of susceptible strains of experimental animals with an encephalitogenic emulsion results in activation of CNS-reactive T cells in lymph nodes that drain the site of immunization [5]. These activated cells leave the lymph nodes and migrate to the CNS, where they are reactivated by local antigen-presenting cells [6]. As a result, the blood–brain barrier is disrupted, and numerous immune cells infiltrate the CNS [7]. Inflammation leads to demyelination and cell death in the CNS, causing detectable neurological sequelae that are observed as clinical signs of EAE. The pathogenesis of CNS-autoimmunity in EAE is considered to be similar to the sequelae underlying the pathogenesis of multiple sclerosis [1], with one important exception: the site of initial activation of CNS-reactive T cells is unknown in multiple sclerosis. Interestingly, the triggering of CNS autoimmunity in multiple sclerosis may occur in the gut through the process of molecular mimicry and/or as a consequence of loss of gut barrier integrity [8–10]. In addition to their potential role in disease initiation, intestinal immune cells are increasingly appreciated as important players in the pathogenesis of multiple sclerosis and EAE [9–14]. Among intestinal immune cells, innate lymphoid cells type 3 (ILC3) have been identified as the prominent regulatory population of relevance for CNS autoimmunity [15]. Moreover, ILC3 modulation through free fatty acid receptor 2 (Ffar2) was shown to have an important role in intestinal immune homeostasis [16, 17]. Thus, in the current study we investigate the contribution of intestinal ILC3 to EAE clinical outcomes and further examine the role of Ffar2 activation to modulate this axis and thereby alter disease progression.

EAE is usually induced in inbred strains of rodents living under strictly controlled environmental conditions. The use of genetically identical animals has the advantage of eliminating genetic variation as a factor in EAE pathogenesis. However, despite the genetic homogeneity of the experimental animals and the controlled environmental conditions, different clinical outcomes are nonetheless regularly observed across the group following immunization. These differential responses to EAE immunization can, therefore, be used to study the mechanisms responsible for limitation or exacerbation of the CNS immune response. We have developed a new subtype of EAE, induced in Dark Agouti (DA) rats with spinal cord homogenate (SCH) without complete Freund’s adjuvant (CFA) [18], and recently characterized it as a reliable multiple sclerosis model which is devoid of neuroimmune-related effects of CFA [19]. Here, we examined the differences in clinical outcomes of rats immunized with SCH with a particular focus on the mechanisms behind the observed differences in lymph nodes draining the site of immunization for both the lamina propria of the intestine as well as in the CNS. The effects of a Ffar2 agonist on intestinal ILC3 in the inductive phase of EAE were studied in the same context.

## Materials and methods

### Experimental animals, EAE

DA rats were obtained from the animal facility of the Institute for Biological Research “Siniša Stanković”. Permission for the experiments was granted by the Veterinary Administration, of the Ministry of Agriculture, Forestry and Water Management, of the Republic of Serbia (N° 323–07-01337/2020–05 and N° 323–07-05815/2020–05/1). Six-month-old female DA rats were immunized with SCH without CFA, monitored, and examined for clinical signs as previously described [19]. The cumulative clinical score (c.s.) is the sum of the daily clinical scores, whereas the mean c.s. is the cumulative c.s. divided by the duration of clinically manifested EAE. Animals that died as a result of EAE received the highest c.s. until the end of the observation period for the presentation of clinical progression Ffar2 agonist (“Cpd1”, compound 1 in patent no. WO 2011/073376 A1) was generously provided by Epics Therapeutics S.A. under Material Transfer Agreement permitting use of the compound [20, 21]. Rats received 15 mg/kg of Cpd1 by oral gavage (0.3 ml), once daily from day of immunization for 6 consecutive days. Distilled water was used to dissolve the agonist and as a vehicle control.

### Histology

Inflammatory infiltrates were detected on spinal cord tissue sections by hematoxylin and eosin (H&E) staining, as previously described [19]. Demyelinating regions in the spinal cord sections were determined by Sudan black staining, as previously described [22]. Quantification of infiltrates and cells per infiltrate and demyelinated regions was performed on 12 spinal cord tissue sections obtained from 3 rats per group (4 sections per rat). ICY Software (BioImage Analysis Lab, Institut Pasteur, Paris, France) was used to quantify demyelination.

Tissue samples of ileum from four animals per group were fixed in 4% phosphate-buffered formaldehyde for 24 h, dehydrated in ethanol (70%, 96% and 100%), cleared in xylol and embedded in paraffin. During embedding, specimens were orientated perpendicular to the long axis of the ileum, so that the entire circumference of the ileal wall was covered when 4–5 µm thick sections were cut. From each specimen, 4 sections were taken and stained according to standard protocol using HE. Sections were photographed using a Leica DM4000 B LED light microscope (Leica, Wetzlar, Germany), and a Leica DFC295 digital camera (Leica, Heerbrugg, Switzerland). The following morphometric parameters were analyzed using ImageJ software [23]: thickness of the mucosa, submucosa and muscularis propria, villus length, depth of the crypt, thickness of the surface epithelium, thickness of the lamina muscularis mucosae and crypt density. Crypt density was calculated as the number of crypts on the longitudinal mucosal sections per 0.1 µm lamina muscularis mucosae (lmm). In addition, each section was carefully examined for the presence of mucosal edema, inflammation and surface epithelial disruption.

### Isolation of cells and cell cultures

Cells of the lymph nodes draining the site of immunization, namely, popliteal lymph node cells (PLNC), as well as spinal cord immune cells (SCIC) were isolated from rats on days 24–28 after immunization, as previously described [19]. Small intestinal lamina propria immune cells (LPIC) were isolated at the same time as PLNC and SCIC according to the protocol described previously [24]. A 40 cm sample of small intestine anterior to the cecum was harvested and cut into pieces (approximately 5 cm long), after which the intestinal contents and Peyer’s patches were removed. The intestine was opened longitudinally, additionally cut into smaller pieces (approximately 1 cm long) and washed thoroughly three times in cold PBS. Samples were then washed with PBS containing 2% FCS and 2.5 mM dithiothreitol (DTT, Sigma-Aldrich) in an orbital shaker (250 rpm, 20 min). Samples were then washed three times in PBS containing 2% FCS and 5 mM EDTA in an orbital shaker (250 rpm, 15 min). Pieces were removed, washed in RPMI 10% FCS (250 rpm, 10 min) and resuspended in a solution of collagenase D (0.2 U/ml) and DNase I (0.1 mg/ml) (both from Roche Diagnostics GmbH, Mannheim, Germany) dissolved in RPMI 10% FCS. Samples were incubated for 45 min at 37 °C in an orbital shaker (350 rpm). After digestion, the tissue was homogenized through a 70 µm cell strainer, collected in PBS 2% FCS 5 mM EDTA and washed twice with the same solution (600 g, 5 min). After centrifugation, the pellet was resuspended in 40% Percoll gradient solution, layered on 80% Percoll, and centrifuged at 700 g for 20 min without rotor acceleration and brake. LPIC were collected at the interface between 40 and 80% Percoll, washed twice in PBS, and resuspended in RPMI 5% FCS for further analysis.

PLNC were grown in RPMI-1640 medium (Capricorn Scientific, Ebsdorfergrund, Germany) supplemented with 2% rat serum, in 24-well plates (5 × 10^6^/ml). PLNC were stimulated with myelin basic protein (10 μg/mL, guinea pig MBP, a kind gift from Professor Alexander Flügel, University of Gottingen, Germany) or myelin isolated from rat spinal cord, as previously described [25]. For myelin extraction, two rat spinal cords were homogenized. Myelin was purified using 0.85 M and 0.32 M sucrose gradients, and myelin sediment was finally resuspended in 500 μl PBS and stored at − 20 °C until further use. PLNC cultures were grown for 48 h, to obtain cell culture supernatants and Treg evidence.

For intracellular cytokine detection, cells were treated for 4 h with a cell stimulation cocktail containing phorbol 12-myristate 13-acetate (PMA), ionomycin and a protein transport inhibitor (Thermo Fisher Scientific, San Diego, CA, USA). All cultivations were performed at 37 °C in a humidified atmosphere (5% CO_2_).

### Cell viability

Cell viability was determined by 3-(4,5-dimethylthiazol-2-yl)-2,5-diphenyltetrazolium bromide (MTT) assay as previously described [26]. Absorbance (540 nm with a correction at 670 nm) was measured using an automated microplate reader (Synergy H1, Agilent BioTek, Santa Clara, CA).

### ELISA

PLNC cell-free culture supernatants were obtained by centrifugation at 500 g for 3 min. The small intestine was dissected and homogenized (1 g/1 ml PBS) using a Dounce homogenizer. The homogenate obtained was centrifuged at 10,000 g (4 °C) for 30 min, and the supernatant was collected. Cytokine concentration in the supernatant was determined by the sandwich ELISA method using MaxiSorp plates (Nunc, Rochild, Denmark) and appropriate capture and detection antibodies for IFN-γ, and IL-17, (Thermo Fisher Scientific, Waltham, MA) and for GM-CSF, IL-1β and IL-6 (R&D Systems, Minneapolis, MN) according to the manufacturer’s instructions. Standard curves were generated with known concentrations of recombinant IFN-γ, and IL-17 (Peprotech, Rocky Hill, NJ), and recombinant GM-CSF, IL-1β and IL-6 (R&D Systems). Absorbance (450 nM, correction at 670 nm) was measured using a Synergy H1multiplate reader. Samples were analyzed in duplicate. The lower and upper detection limits were 30 pg/mL and 10 ng/mL, respectively.

### Cytofluorimetry

Cells were stained with the following antibodies: eFluor450-conjugated anti-CD45 (monoclonal mouse OX1, Thermo Fisher Scientific), FITC- or PE-conjugated anti-CD4 (monoclonal mouse OX35, Thermo Fisher Scientific), FITC-conjugated anti-CD3 (monoclonal mouse G4.18, Thermo Fisher Scientific), FITC-conjugated anti-CD45RA (monoclonal mouse OX33, Thermo Fisher Scientific), FITC-conjugated anti-IFN-γ (polyclonal rabbit, Thermo Fisher Scientific), PE-conjugated anti-MHC class II (monoclonal mouse HIS19, Thermo Fisher Scientific), PE-conjugated anti-CD25 (monoclonal mouse OX39, Thermo Fisher Scientific), PE-conjugated anti-GATA-3 (monoclonal rat TWAJ, Thermo Fisher Scientific), PerCP-Cy5.5-conjugated anti-IL-17 (monoclonal rat eBio17B7, BD Biosciences, San Jose, CA), PerCP-Cy5.5-conjugated anti-T-bet (monoclonal mouse 4B10, Thermo Fisher Scientific), PerCP-Cy5.5-conjugated anti-Foxp3 (monoclonal rat FJK-16 s, Thermo Fisher Scientific), AF647-conjugated anti-IL-10 (monoclonal mouse A5-4, BD Biosciences), AF647-conjugated anti-Ki67 (monoclonal mouse B56, BD Biosciences), APC-conjugated anti-CD40 (monoclonal Armenian hamster HM40-3, Thermo Fisher Scientific), APC-conjugated anti CD134 (monoclonal mouse OX40, Thermo Fisher Scientific), eFluor710-conjugated anti-Rorγt (monoclonal rat AFKJS-9, Thermo Fisher Scientific), and PE-Cy7-conjugated anti-Eomes (monoclonal rat Dan11mag, Thermo Fisher Scientific). FITC-conjugated Annexin V (Biolegend, San Diego, CA) was used for apoptosis detection. Intracellular staining for cytokines and FoxP3 was performed with Foxp3/transcription factor fixation/permeabilization concentrate and diluent, intracellular fixation and permeabilization buffer set, and permeabilization buffer (all from Thermo Fisher Scientific), according to the manufacturers recommended procedure. Appropriate isotype control antibodies were used as needed to establish gates for cell marker positivity. Samples were collected using a CytoFLEX flow cytometer (Beckman Coulter, Indianapolis, IN) and analyzed using CytExpert software (Beckman Coulter). Cytofluorimetry results are presented as the percentage of cells bound by an appropriate antibody. Gating strategies are shown in the (Additional file 1: Figs. S1–S4).

### Gut permeability

To determine gut permeability, rats were starved overnight and then treated with BSA–FITC (INEP, Belgrade, Serbia) dissolved in water (oral administration, 100 mg/rat). The rats were left without access to water for 2 h, and then blood was collected. Serum was obtained by centrifugation at 2000 rpmi for 10 min in a MiniSpin centrifuge. Serum was pipetted in triplicate into 96 well optical bottom plates (Nunc), (50 μl/well) to measure fluorescence intensity (f.i.) using a Synergy H1 multi-plate reader (excitation at 485 nm/emission at 530 nm).

### Immunoblot

A 1 cm piece of long ileum was opened horizontally and washed in ice-cold PBS. Approximately 100 mg of ileum tissue was frozen and stored at − 80 °C, until use. To obtain the whole cell protein extract, the tissue was homogenized on ice with a teflon borosilicate glass homogenizer in 10 volumes of ice-cold RIPA buffer (50 mM Tris–HCl, pH 7.5, 150 mM NaCl, 1% NP-40, 0.1% SDS, 0.5% Triton X-100, 1 mM EDTA, 1 mM EGTA) with protease inhibitor mix G (Serva, Heidelberg, Germany), 0.1 mM phenylmethylsulphonyl fluoride, 2 mM dithiothreitol, 3 mM benzamidine, 5 mM sodium pyrophosphate and 2 mM sodium vanadate, with 20 up-and-down strokes. The homogenate was sonicated on ice for 3 × 5 s and then extracted at 0 °C for 30 min. After centrifugation at 16000 g at 4 °C for 30 min, the supernatants were collected and stored at − 80 °C. The protein concentration of supernatants was determined by Lawry assay using bovine serum albumin (BSA) as standard.

Samples were mixed with equal volume of 2 × SDS-sample buffer (denaturing buffer) and boiled for 3 min. Samples (40 µg protein/well) were separated in a 10% SDS–polyacrylamide gel and transferred to Immobilion-FL PVDF membrane (Merck Millipore, Burlington, MA) using the Mini Trans-blot module (Bio-Rad Laboratories, Hercules, CA). Membranes were blocked with 5% nonfat dry milk in phosphate buffered saline for 1 h at room temperature, and incubated with Thermo Fisher Scientific primary rabbit polyclonal antibodies: occludin (40–4700; 1:1000), ZO-1 (61–7300; 1:1000), and anti-β-actin (PA1-183; 1:2000) which was used as loading control, overnight at 4 °C in blocking solution (2% BSA in PBS). Blots were developed using an HRP-linked anti-rabbit IgG antibody (#7074, Cell Signaling Technology; 1:2000).

Densitometry of protein bands was determined using the Image J Imaging System and expressed as relative values.

### Statistical analysis

K-means cluster analysis (IBM SPSS Statistics for Windows, Version 25.0. Armonk, NY: IBM Corp) was used to divide the rats into three groups classified as mild, moderate, and severe. The variables selected for cluster analysis were day of onset, cumulative clinical score, mean clinical score, and maximal clinical score. The fourth group (lethal) consisted of terminally ill rats. The sample size for further analyzes was determined based on our previous studies, using Biomath® Power Calculator (http://biomath.info/power). The clinical variables were tested using Pearson's r-correlations. Statistical analysis was performed using GraphPad Prism 9 software (GraphPad Software, San Diego, California, USA). The significance of differences between groups was determined using a two-tailed Student’s *t* test or a one-way test (ANOVA) followed by a Tukey post hoc test, as indicated in the figure legends. A *p* value of less than 0.05 was considered statistically significant.

## Results

### Clinical outcomes in SCH-immunized rats

**Fig. 1:**
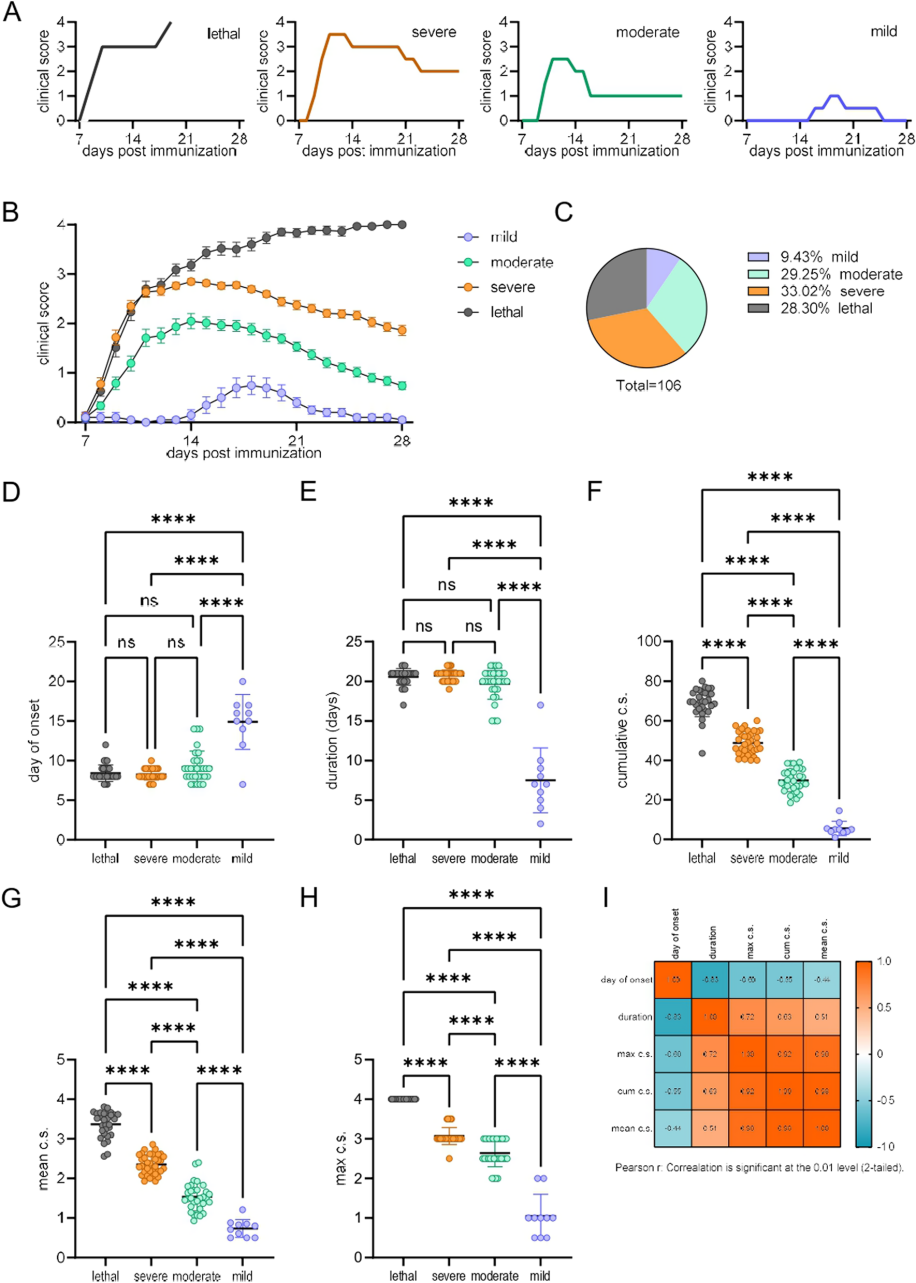
Different EAE outcomes in SCH-immunized DA rats. DA rats were immunized with SCH and scored daily for clinical signs. **A** Representative plots of clinical outcomes are presented. **B** Grouped clinical outcomes. **C** Distribution of rats among groups (*n* = 106). **D**–**H** Parameters of EAE. **I** Correlation matrix of EAE parameters (*n* = 106). Data are presented as mean ± SEM (**B**) or mean ± SD (**D**–**H**) from 10 (mild), 31 (moderate), 35 (severe), and 30 (lethal) obtained in 6 independent experiments. *p < 0.05, ****p < 0.001, ns—not significant

Immunization of rats with SCH resulted in variable EAE clinical outcomes (Fig. 1A, B). The minority of immunized rats developed mild EAE, with a maximum clinical score of 1.5 or less (“mild”), and showed no clinical signs by 28 d.p.i. (Fig. 1C). The rest of the rats developed overt disease, with a maximum c.s. of 2–3.5. Some of the rats died as a result of the disease (“lethal”). The others survived and had a c.s. of 2 or higher (“severe”), or c.s. 1.5 or less (“moderate”) on day 28 d.p.i. It is important to note that the results presented here are from six independent experiments, and that a similar distribution was observed in each of these experiments. Comparison of basic clinical parameters among groups showed that there were no significant differences in the day of onset (Fig. 1D) nor on the duration of EAE (Fig. 1E), with the exception of the mild group, in which EAE started later and lasted for a shorter period of time. The cumulative c.s. (Fig. 1F), mean c.s. (Fig. 1G), and maximum c.s. (Fig. 1H) differed significantly among the four groups. Significant correlations among EAE parameters was determined and presented as a correlation matrix (Fig. 1I).

**Fig. 2:**
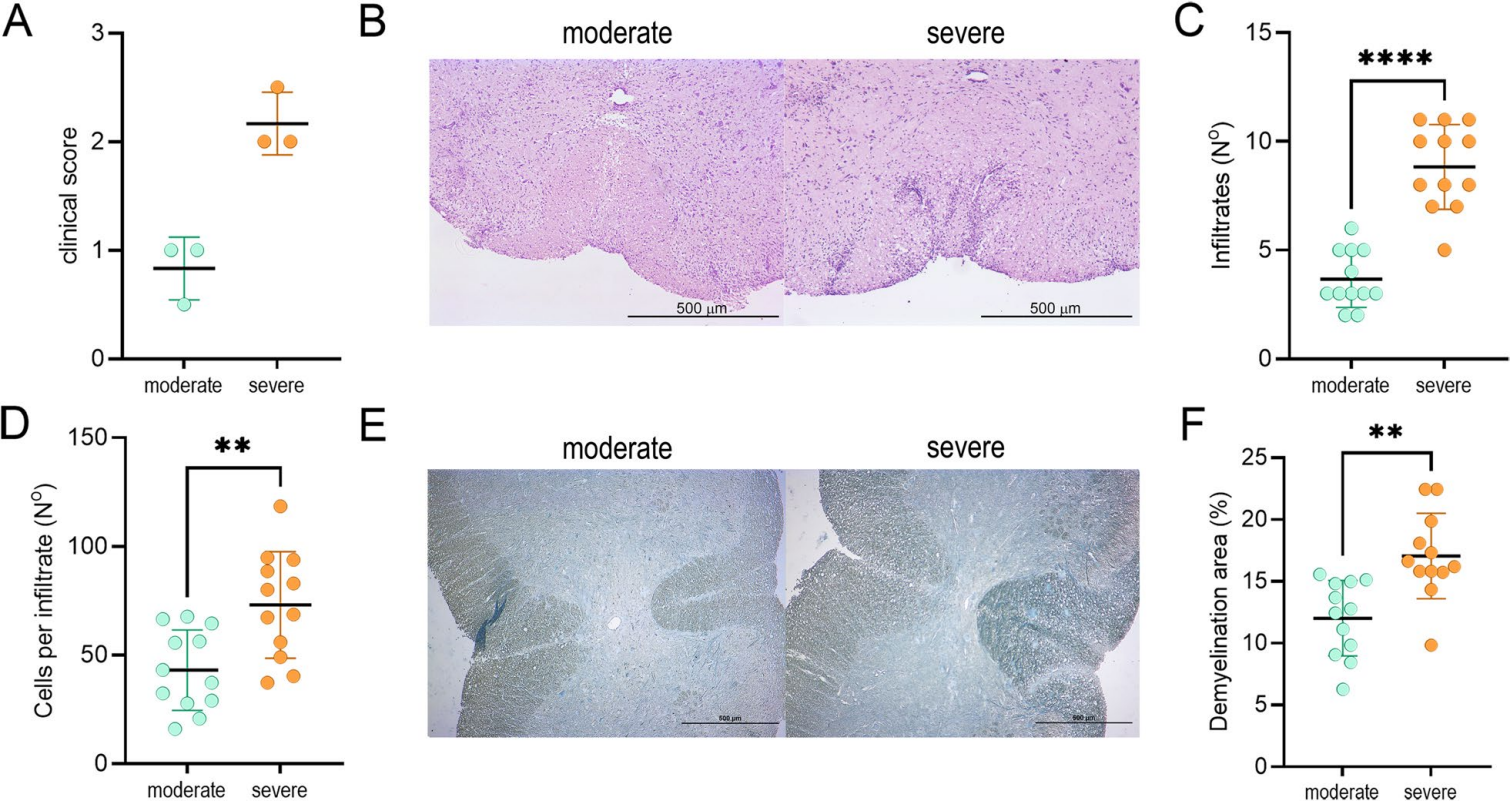
Histological comparison of spinal cords from DA rats with different clinical outcomes. DA rats were immunized with SCH and sacrificed at 24–28 dpi. **A** Clinical scores at the time of spinal cord isolation. **B** Representative sections of spinal cord H&E staining. **C** Number of infiltrates per spinal cord section (*n* = 12). **D** number of cells per infiltrate (*n* = 12) **E** Representative sections of spinal cord Sudan black staining. **F** Quantification of demyelination (*n* = 12). Data are presented as mean ± SD (**A**, **C**, **D**, **F**). **p < 0.01, ****p < 0.001

Next, we focused on the differences between the severe and moderate group in rats. Spinal cords were isolated in the severe and moderate groups at the time when their clinical scores differed significantly, i.e., at 24–28 dpi (Fig. 2A). In the late phase of EAE, there were more infiltrates at SC and more cells per infiltrate in the severe group than in the moderate group (Fig. 2B–D). Demyelination was also more pronounced in the severe group (Fig. 2E, F).

### Comparison of SCIC composition in moderate and severe EAE

**Fig. 3:**
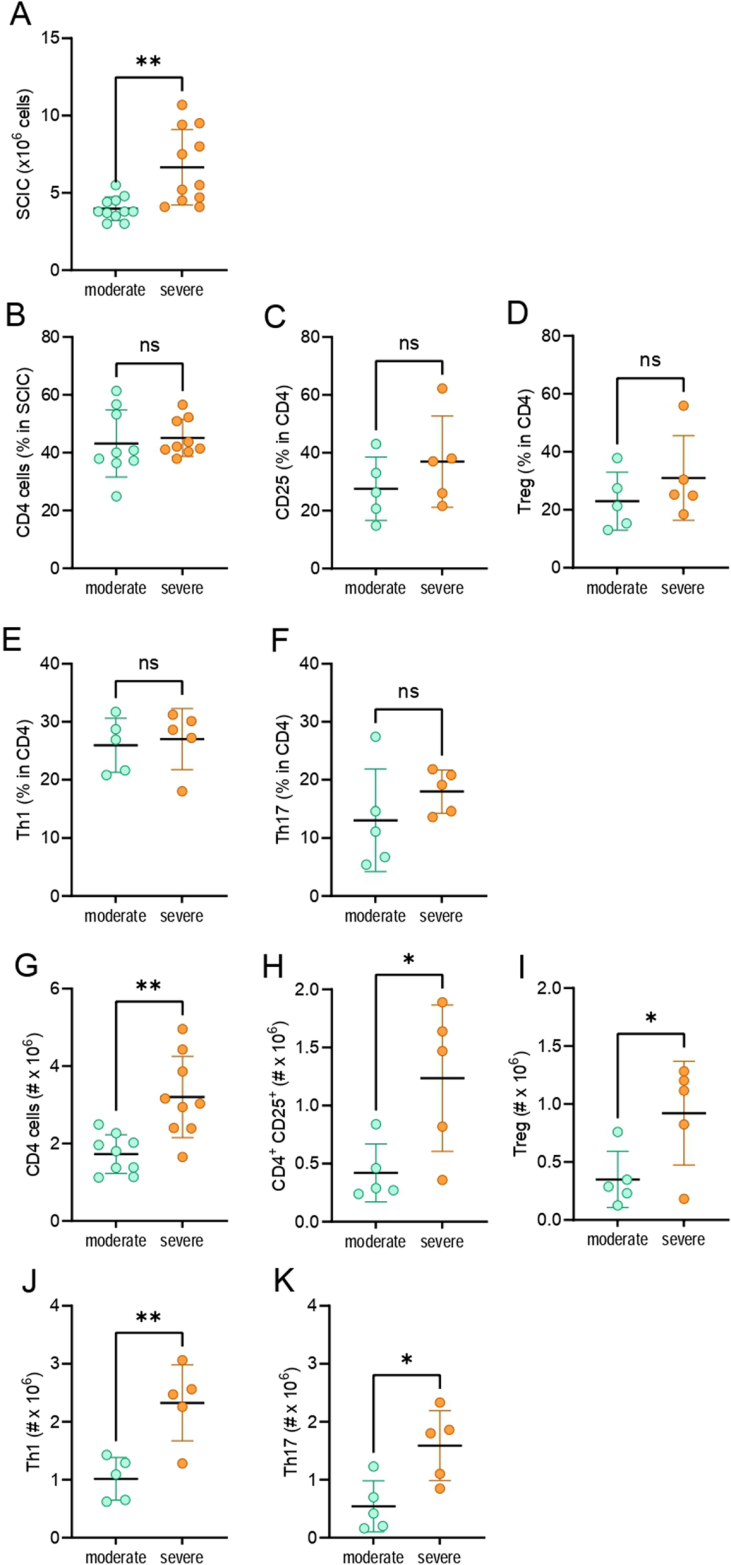
Composition of SCIC in the moderate and severe groups. DA rats immunized with SCH were sacrificed on days 24–28 p.i. SCIC were isolated and counted (**A**). Percentage (**B**–**F**) and cell number (**G**–**K**) of cell populations were determined by flow cytometry. Data are expressed as mean ± SD (*n* = 5–11, from 2 to 4 independent experiments). *p < 0.05, **p < 0.01, ns—not significant

To determine whether the different clinical courses in the moderate and severe groups correspond to a different number and/or composition of immune cell infiltrates in the CNS, SCIC were isolated 24–28 dpi and analyzed by flow cytometry. A higher number of SCIC was observed in the severe group (Fig. 3A). No statistical difference was observed in the proportion of CD4^+^ T cells, activated CD4^+^ T cells (CD25^+^), Treg (CD4^+^CD25^+^FoxP3^+^), IFN-γ^+^, and IL-17^+^ cells among CD4^+^ cells (Fig. 3B–F). However, absolute numbers of CD4^+^ T cells, activated CD4^+^ T cells, Treg, Th1 (CD4^+^IFN-γ^+^), and Th17 (CD4^+^IL-17^+^) cells were higher in the severe group and showed statistical difference (Fig. 3G–K).

Also, no difference was observed in the percentage of CD8^+^ T cells, B cells (CD45RA^+^), and MHC class II^+^ B cells between the groups (Additional file 1: Fig. S5A–C). Nevertheless, a statistically higher absolute number of B cells, but not CD8^+^ T cells and MHC class II^+^ B cells was observed in the severe group (Additional file 1: Fig. S5D–F).

To examine whether the observed differences in SCIC could be explained by prolonged reactivity of PLNC to CNS antigens, PLNC obtained from moderate and severe rat groups on days 24–28 d.p.i. were challenged with MBP or myelin in vitro. There was no difference in the cellularity of PLNC (Additional file 1: Fig. S6A) nor on the release of IFN-γ in response to MBP or myelin between the groups (Additional file 1: Fig. S6B, C). The proportion of activated CD4^+^ T cells (CD25^+^) and Treg cells among PLNCs challenged with myelin also did not differ between groups (Additional file 1: Fig. S6D–G). Collectively, these data do not indicate that prolonged reactivity of PLNC is the basis of the sustained differences observed in the SCIC.

**Fig. 4:**
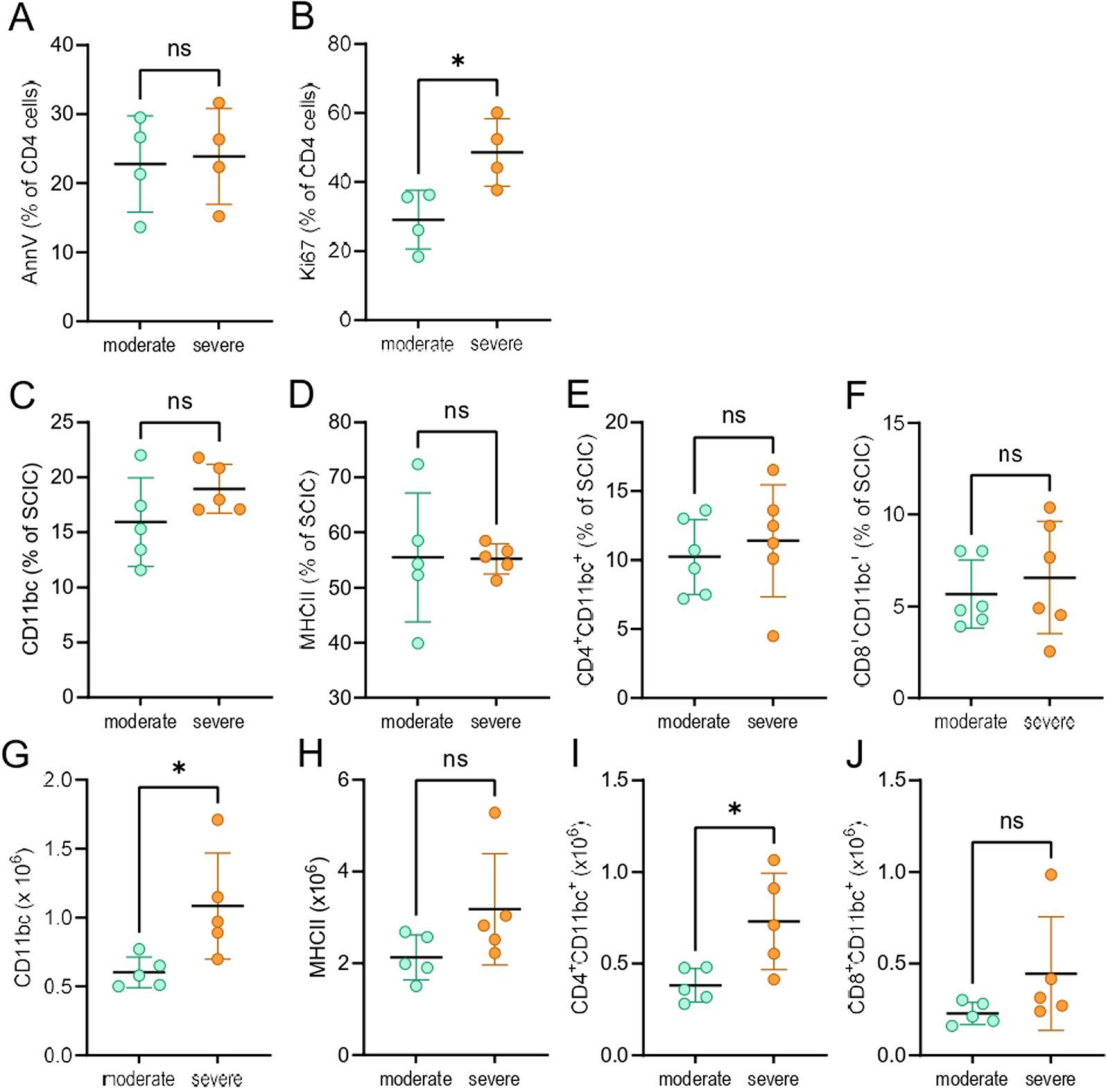
Apoptosis and proliferation of CD4 SCIC in the moderate and severe group. DA rats immunized with SCH were sacrificed on days 24–28 p.i. SCIC were isolated and percentage (**A**–**F**) and cell number (**G**–**J**) of cell populations were determined by flow cytometry. Data are expressed as mean ± SD (*n* = 4–6, from 2–3 independent experiments). *p < 0.05, ns—not significant

To investigate the reason for the higher number of CD4^+^ T cells in the spinal cord of the severe group, apoptosis and proliferation of these cells were determined. While there was no statistically significant difference in the proportion of apoptotic cells among CD4^+^ T cells (Fig. 4A), percentage of proliferating CD4^+^ T cells was higher in the severe group (Fig. 4B). Furthermore, there was more CD11bc^+^ cells, as driven by an increase in the subset of CD4^+^CD11bc^+^ cells but not of CD8^+^CD11bc^+^ cells, whereas there was no difference in MHC class II^+^ cells in the severe group (Fig. 4C–J). Notably, in all of the examined CD11bc^+^populations, the proportion of MHC class II^+^ cells was higher than 98%. Thus, an increased proliferation of CD4^+^ T cells in the spinal cord, as a consequence of enhanced antigen-presenting activity of CD4^+^CD11bc^+^ macrophages/microglia, might contribute to the higher number of these cells in the spinal cord of the severe group.

### Comparison of inflammatory markers in the gut in moderate and severe EAE

**Fig. 5:**
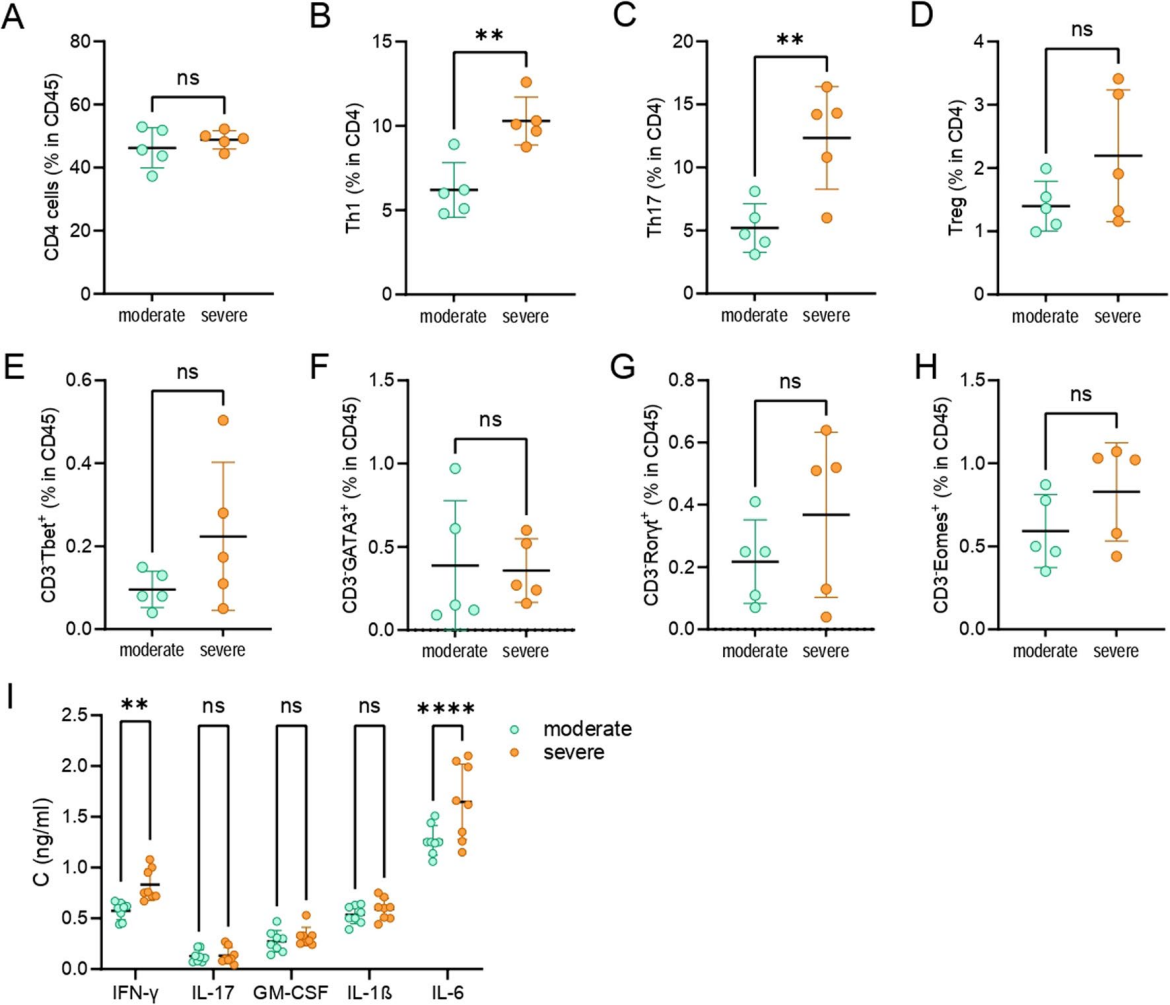
LPIC composition in the moderate and severe group. DA rats immunized with SCH were sacrificed at days 24–28 p.i. and LPIC (**A**–**H**) or small intestine (**I**) were isolated. Percentage of cell populations was determined by flow cytometry (**A**–**H**). Cytokine levels in small intestinal homogenate supernatants were determined by ELISA (**I**). Data are presented as mean ± SD (*n* = 5 or 8, from 2–3 independent experiments). **p < 0.01, ****p < 0.001, ns—not significant

The composition of the LPIC was also analyzed to evaluate whether differences in the gut immune system correlated with the clinical outcomes that distinguished the severe and moderate groups. There was no difference in the proportion of CD4^+^ cells between the moderate and severe groups (Fig. 5A), but a higher proportion of Th1 and Th17 was observed in the severe group (Fig. 5B, C). In addition, no differences in the proportion of Treg, ILC1/NK (CD45^+^CD3^−^T-bet^+^ or CD45^+^CD3^−^Eomes^+^), ILC2 (CD45^+^CD3^−^GATA-3^+^), and ILC3 (CD45^+^CD3^−^Rorγt^+^) were observed between groups (Fig. 5D–H), although higher levels of IFN-γ and IL-6 (but not of IL-17, GM-CSF and IL-1β) was determined in supernatants of small intestine homogenates of rats belonging to the severe group (Fig. 5I).

**Fig. 6:**
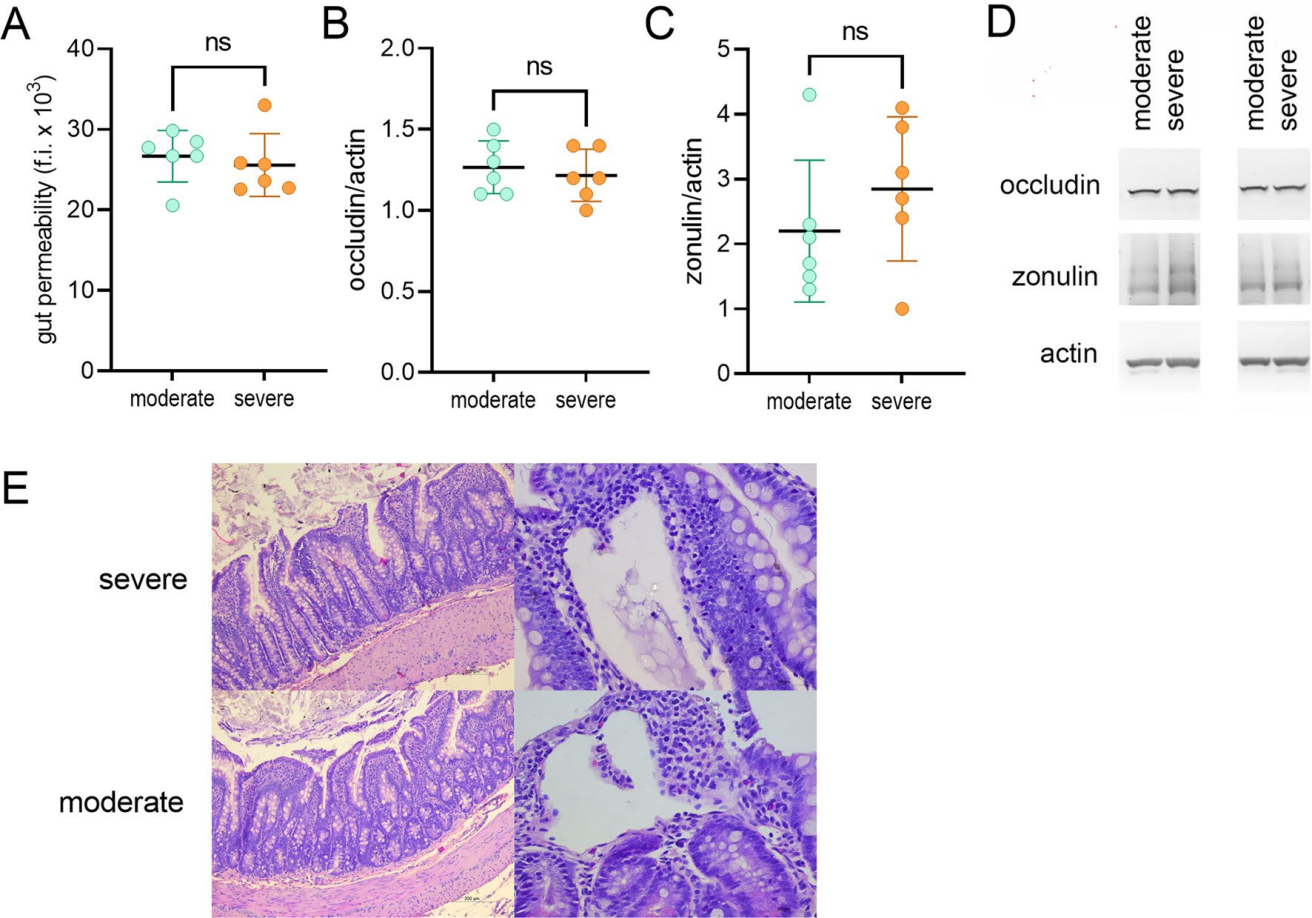
Gut permeability and small intestine histology in the moderate and severe groups. DA rats were immunized with SCH and analyzed at days 24–28 post immunization. **A** Rats were orally administered BSA–FITC and their plasma samples were collected 2 h later. Fluorescence intensity (f.i.) was detected in the plasma samples. **B**, **C** Quantification of the immunoblot of the small intestine samples. **D** A pair of representative immunoblots. **E** Histology of the ileum, HE stained section. Section through all layers of the ileum wall and ectatic lymphatic vessels in the lamina propria. Data are presented as mean ± SD (*n* = 6, from 3 independent experiments). ns—not significant

There was no difference in gut permeability between the groups at 24–28 d.p.i. (Fig. 6A). Contemporaneous immunoblot analysis of the expression of the tight junction proteins occludin and zonulin in the small intestine showed no difference between the severe and moderate groups (Fig. 6B–D, Additional file 1: Fig. S7). Histological analysis of the gut also showed no significant differences between the groups (Fig. 6E). Various histopathologic changes were observed in the ileum of both the moderate and severe animals: variable elongation of crypts, focal branched crypts, focal villous blunting, focal scattered eosinophils in the lamina propria and mild depletion of goblet cells in the surface epithelium. There was also varying degrees of mucosal edema present, in some sections with extremely dilated, ectatic lacteals in both groups (Fig. 6E). There were no significant differences in any of the morphometric parameters analyzed (Table 1).

**Table 1:** Morphometric analysis of ileum in severe and moderate EAE rats

	Severe	Moderate
Mucosal thickness (µm)	395.0 ± 72.3	405.0 ± 84.2
Submucosal thickness (µm)	22.8 ± 9.6	25.0 ± 8.5
Muscularis propria thickness (µm)	78.2 ± 34.0	80.8 ± 53.6
Lamina muscularis mucosae thickness (µm)	16.7 ± 3.0	16.0 ± 4.3
Villus length (µm)	238.4 ± 90.0	216.1 ± 77.0
Crypt depth (µm)	219.9 ± 56.3	219.8 ± 96.6
Crypt density (per 0.1 µm^2^ lmm)	1.7 ± 0.3	1.9 ± 0.4
Surface epithelium thickness (µm)	16.8 ± 3.3	15.9 ± 4.3

*n* = 4, from 2 independent experiments

### Effect of FFAR2 agonist on EAE in SCH-immunized rats

**Fig. 7:**
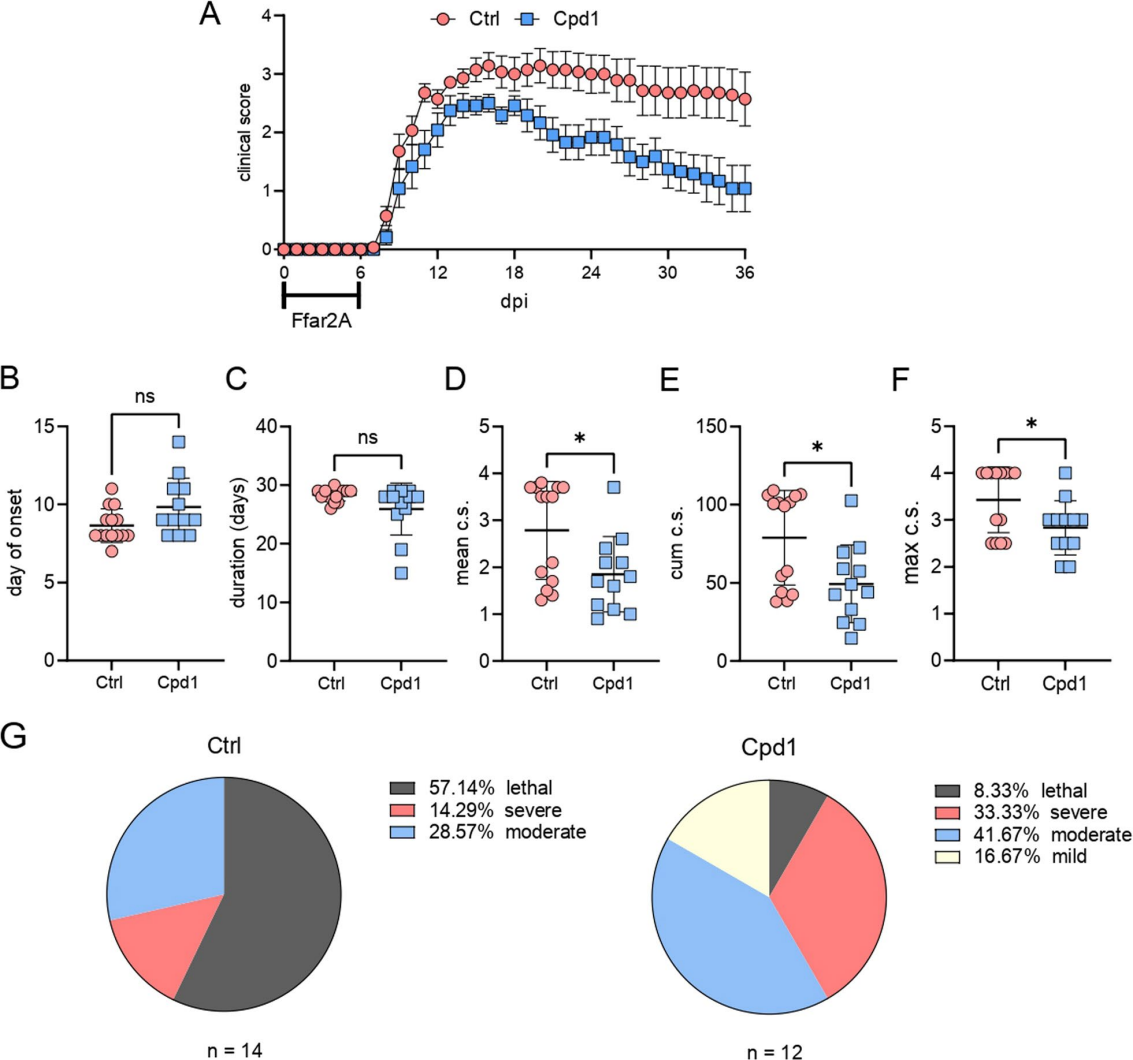
Effects of Ffar2 agonist Cpd1 on EAE. DA rats were immunized with SCH and treated orally with Cpd1 (15 mg/kg, q.d., *n* = 12) or vehicle (water, *n* = 14) 0–5 d.p.i. Clinical scores were monitored daily and presented as mean ± SEM (**A**). Clinical parameters of EAE observed in a single experiment are presented as mean ± SD (**B**–**F**). Distribution of rats among clinical groups was determined on day 28 d.p.i. (**G**). *p < 0.05, ns—not significant

To investigate whether modulation of Ffar2 in the inductive phase of EAE is relevant to the clinical expression of disease, the Ffar2 agonist (Cpd1) was dosed orally to rats beginning on the day of immunization for 6 consecutive days. Administration of the agonist significantly reduced the clinical signs of EAE (Fig. 7A). While there was no difference between the groups in the day of onset and duration of EAE (Fig. 7B, C), cumulative, mean and maximum c.s. were reduced in the Cpd1 group (Fig. 7D–F). Moreover, there was a shift in the distribution from the lethal and severe to the moderate and mild group in the rats treated with Cpd1 (Fig. 7G).

**Fig. 8:**
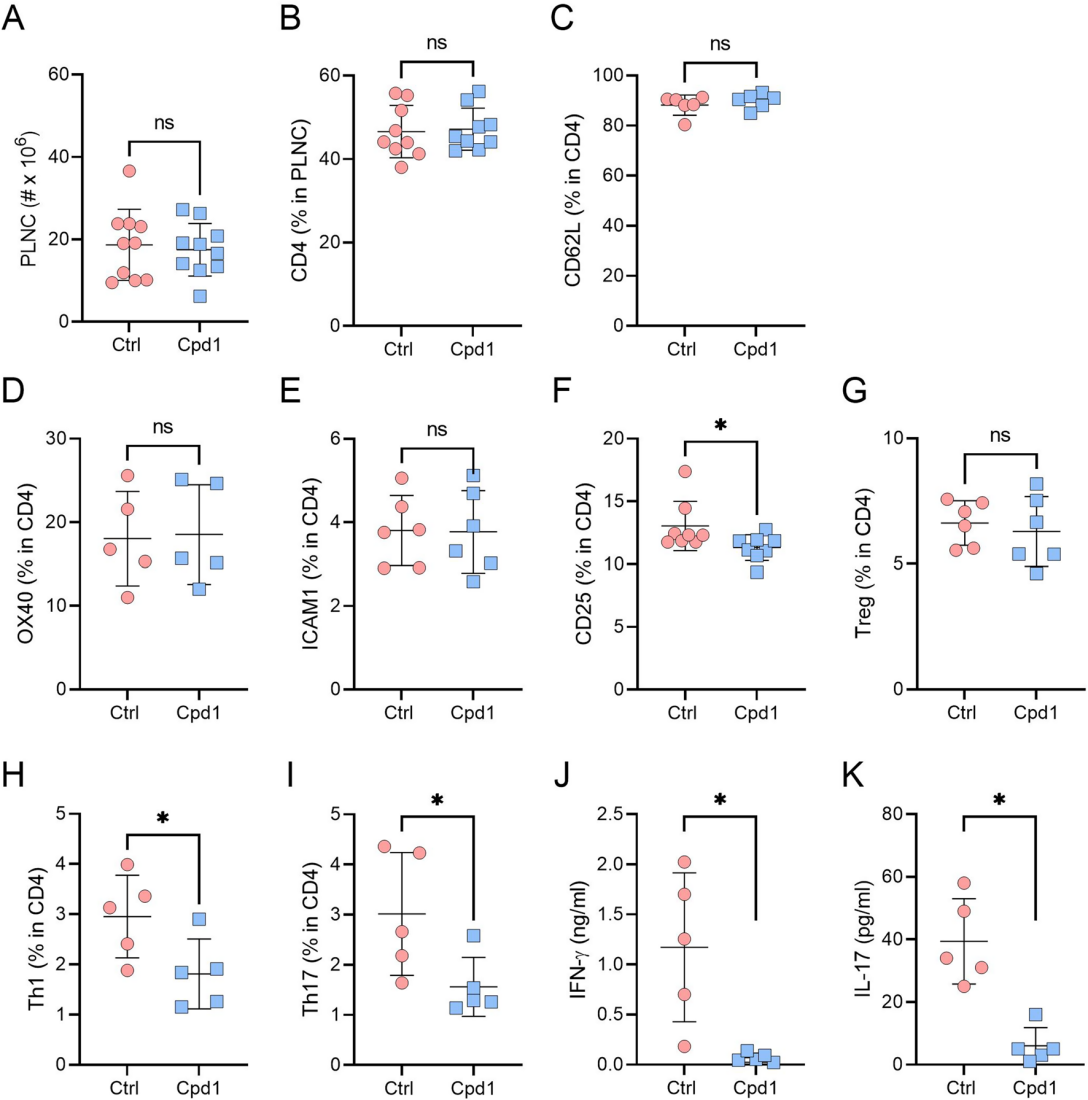
Effects of Cpd1 on PLNC. DA rats were immunized with SCH and treated orally with Cpd1 (15 mg/kg, q.d.) or vehicle (water) 0–5 d.p.i. Subsequently, PLNC were isolated and counted (**A**). Percentage of cell populations was determined by flow cytometry (**B**–**I**). PLNC were exposed to MBP for 48 h and IFN-γ and IL-17 levels in cell culture supernatants were determined by ELISA (**J**, **K**). Data are presented as mean ± SD (*n* = 5–10, from 2 to 4 independent experiments). *p < 0.05, ns—not significant

There were no differences in the cellularity of PLNC, nor in the proportion of CD4^+^ T cells, naïve CD4 + T cells (CD62L^+^), activated CD4^+^ T cells (OX40^+^, ICAM1^+^, CD25^+^) and Treg cells (Fig. 8A–G) between Cpd1-treated and untreated rats on day 5 post immunization. However, the proportion of Th1 and Th17 cells was reduced in PLNC corresponding to a moderated activity of PLNC to produce IFN-γ and IL-17 in response to MBP (Fig. 8H–K).

**Fig. 9:**
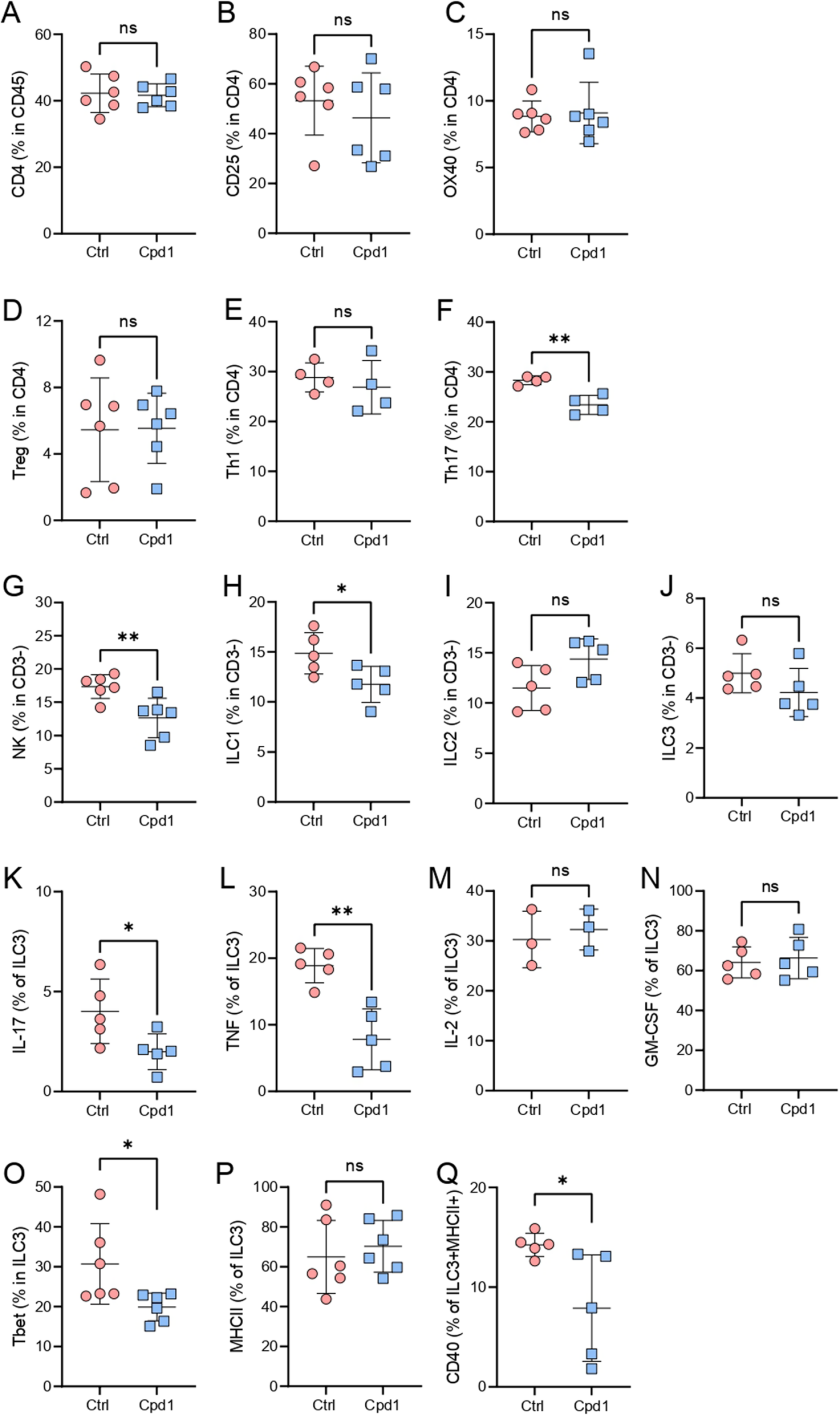
Effects of Cpd1 on LPIC. DA rats were immunized with SCH and treated orally with Cpd1 (15 mg/kg, q.d.) or vehicle (water) 0–5 d.p.i. Subsequently, LPIC were isolated. Percentage of cell populations was determined by flow cytometry. Data are presented as mean ± SD (*n* = 3–6, from 2 to 4 independent experiments). *p < 0.05, **p < 0.01, ns—not significant

Cpd1 treatment did not affect the proportion of CD4^+^ T cells, nor of activated CD4^+^ T cells (CD25^+^ or OX40^+^), nor of Treg and Th1, but it reduced the proportion of Th17 in small intestine lamina propria (Fig. 9A–F). Furthermore, Cpd1 treatment led to a significant reduction in the number of NK cells and ILC1 (Fig. 9G, H), but not of ILC2 and ILC3 (Fig. 9I, J). However, Cpd1 treatment did alter the profile of ILC3 cells as fewer ILC3 cells produced IL-17 and TNF (Fig. 9K, L), but without concomitant changes in the proportion of ILC3 cells producing IL-2 and GM-CSF (Fig. 9M, N);there were also fewer Tbet^+^ cells within the ILC3 population (Fig. 9O). Finally, Cpd1 treatment did not alter the proportion of MHC class II-expressing ILC3 (Fig. 9P), but significantly reduced the percentage of CD40^+^ cells among MHC class-expressing ILC3 (Fig. 9Q).

## Discussion

In this work, we show that immunization of DA rats with SCH produces four different types of clinical expression of EAE: lethal and mild at the extremes of this diversity, and two other groups in which the rats develop severe neurological deficits, and then either recover or do not recover to a c.s. 1.5 or less on days 24–28 after immunization. Importantly, the rats that do not recover (severe) have more abundant infiltrates of immune cells, particularly T cells in the CNS at the timepoints indicated above compared with the rats that do recover (moderate). At the same time, a higher proportion of Th1 and Th17 cells and increased levels of IFN-γ and IL-6 are observed in the small intestine of rats with severe EAE. Notably, we demonstrate that modulation of immune cells in the small intestine and in lymph nodes draining the site of immunization by oral application of a Cpd1 in the inductive phase of the disease protects against the clinical severity of EAE as exemplified by the shifted distribution of clinical subtypes towards milder forms.

Much of our current work is devoted to the small intestine which is increasingly recognized as a major player in CNS autoimmunity. Indeed, the lamina propria of the gut, and the gut-associated lymph nodes are among the possible candidates for the site of initial CNS-reactive T-cell activation by molecular mimicry in humans [8]. Reactivity of encephalitogenic cells against various antigens of gut microorganisms has been demonstrated in multiple sclerosis and EAE [9, 10, 14]. Similarly, the gut microbiota of multiple sclerosis patients has been shown to provoke EAE in germ-free mice [27]. Finally, it has been reported that encephalitogenic cells migrate to the gut, where they can be supported in their autoreactivity or converted into regulatory cells [11–13]. Thus, modulation of the immune response in the gut likely influences immune activity in lymph nodes draining the site of immunization and in the CNS of EAE animals. Elevated proportion of Th1 and Th17 cells, and increased levels of IFN-γ and IL-6 in the small intestine observed in the severe group compared with the moderate group are consistent with a previous report showing increased numbers of Th1 and Th17 cells in the gut lamina propria, Peyer’s patches, and mesenteric lymph nodes of mice with EAE, both before the onset of clinical symptoms, and at the peak of disease [28]. In addition, this report demonstrated increased intestinal permeability, alterations in tight junction function, and altered intestinal morphology, i.e., clear evidence of intestinal barrier disruption [28]. However, we were unable to detect differences in gut barrier permeability or expression of the major tight junction proteins, zonulin and occludin, between the moderate and severe groups. A possible explanation for this discrepancy may be the different timepoints of the investigation in the two studies. It will certainly be important to determine whether intestinal barrier integrity at earlier timepoints is of importance to clinical outcomes in SCH-immunized DA rats. Similarly, evaluation at earlier timepoints may be warranted to contextualize any temporal differences in immune cell populations of lamina propria (e.g., Treg, ILC1, ILC2, ILC3, NK cells) as changes in these immune cell populations were not observed in the later timepoints recorded in this report.

This late sampling time may also explain the observation that there is no difference in PLNC response to CNS antigens in this work. Again, earlier timepoints may be more appropriate to determine the relationship between the strength of T-cell reactivity against CNS antigens in lymph nodes draining the site of immunization and clinical outcomes of EAE. Therefore, it is an essential requirement to identify early biomarkers that could serve as predictors of the clinical course of EAE. It has already been shown that differences in the clinical expression of passive EAE in rats depends upon the antigen specificity of CNS reactive T cells [29]. Considering that rats are immunized with SCH, one can expect a T-cell response to different CNS antigens. So far, we have shown that T cells from SCH-immunized rats respond to MBP [19]. However, reactivity to other CNS antigens has not been tested. Therefore, it will be important to determine the antigen specificity of encephalitogenic T cells in our model and to find out if dominant antigen specificity determines the intensity of EAE in SCH-immunized DA rats.

The Ffar2 agonist Cpd1 was previously shown to selectively act upon ILC3 to promote gut immune homeostasis in mice [16]. Importantly, Ffar2 modulation did not affect lymphocytes of adaptive immunity, nor ILC1 or ILC2, but potentiated ILC3 proliferation and IL-22 production as the basis for the mechanism of action for the observed protective effect of Cpd1 against colitis and *Citrobacter rodentium* infection [16]. Similarly, it has also been shown that Ffar2-mediated potentiation of ILC3 activity to produce IL-22 was beneficial to ameliorating *Clostridium difficile* infection [17]. Recently, it has been reported that colonic intraepithelial cells treated with a different Ffar2 agonist (4-CMTB) in vitro and then adoptively transferred into EAE recipient mice during the inductive phase of the disease efficiently ameliorated EAE [30]. In our study, we build upon these previous findings by demonstrating that dosing of Cpd1 during the inductive phase of EAE significantly reduces the severity of disease. In the work of Prado and colleagues [30], the focus of the study was on the influence of the Ffar2 agonist 4-CMTB on colonic T cells. They were able to demonstrate that encephalitogenic T cells migrate into the colonic intraepithelial compartment before they infiltrate CNS. Furthermore, transfer of 4-CMTB-treated intraepithelial cells into EAE mice led to retention of encephalitogenic cells in the gut mucosa, and consequently prevented their infiltration into CNS [30]. This study delineates the importance of Ffar2 signaling in colonic T cells for counteracting CNS autoimmunity. In comparison, we have examined the in vivo effects of Cpd1 treatment on small intestine and gut-resident ILC populations as it relates to clinical outcomes in the EAE model in a manner complementary to the study of Prada and colleagues in defining how Ffar2 agonism effectively modulates CNS autoimmunity.

Here, the effect of the Ffar2 agonist Cpd1 to reduce disease severity in EAE corresponded to its effects to reduce the population of small intestine lamina propria ILC3 that produced major proinflammatory cytokines, IL-17 and TNF. Indeed, IL-17 originating from ILC3 has previously been shown to contribute to high salt-induced intestinal inflammation [31]. Moreover, ILC3 seem to be the major producer of TNF in the intestine [32]. Cpd1 did not affect the populations of IL-2 and GM-CSF-expressing ILC3. It was previously shown that IL-2 produced by ILC3 provided crucial support to Treg in intestine [33], while GM-CSF potentiated tolerogenic properties of dendritic cells [34]. This shift in the balance between pro- and anti-inflammatory ILC3 profiles with Cpd1 treatment steering towards the latter would be expected to potentiate the regulatory environment in the gut, affecting encephalitogenic properties of T cells arriving into the gut as a part of EAE pathogenesis [11–13, 15]. Furthermore, the finding that Cpd1 treatment inhibited the expression of co-stimulatory molecule CD40 by MHC class II^+^ ILC3 could also contribute to the restraint of the encephalitogenic response. Indeed, it was shown that MHC class II antigen presentation by ILC3, but without proper co-stimulatory signals, directed T-cell differentiation towards Treg [35, 36]. Finally, conversion of ILC3 to ILC1 occurs in inflammation, and it can be detected by expression of T-bet [37]. Thus, the observed decrease in proportion of T-bet expressing intestinal ILC3 under the influence of Cpd1 is an additional mechanism by which ILC3 may be directed towards an anti-encephalitogenic mode in the context of the CNS autoimmunity. In total, these findings indicate that Ffar2-mediated changes in the immunomodulatory profile of ILC3 cells in the lamina propria of the small intestine are the basis of the improved clinical outcomes in response to Cpd1 treatment in EAE.

Cpd1 was previously reported to act specifically on gut-resident ILC3 cells as the basis for its regulatory effects on gut immunity [16]. Here, we show that Cpd1 activation of the regulatory activity of ILC3 cells elicits a reduced encephalitogenic Th1/Th17 response in the lymph nodes draining the site of immunization. These findings correspond with the previous demonstration, using endoscopic photoconversion, that both myeloid and lymphoid populations can migrate from the intestine to distant lymph nodes and spleen [38] thereby supporting the concept that a regulatory immune response local to the gut can be transferred to distant lymphoid sites, such as popliteal lymph nodes draining the site of EAE immunization as in the model used in this investigation.

## Conclusions

**Fig. 10:**
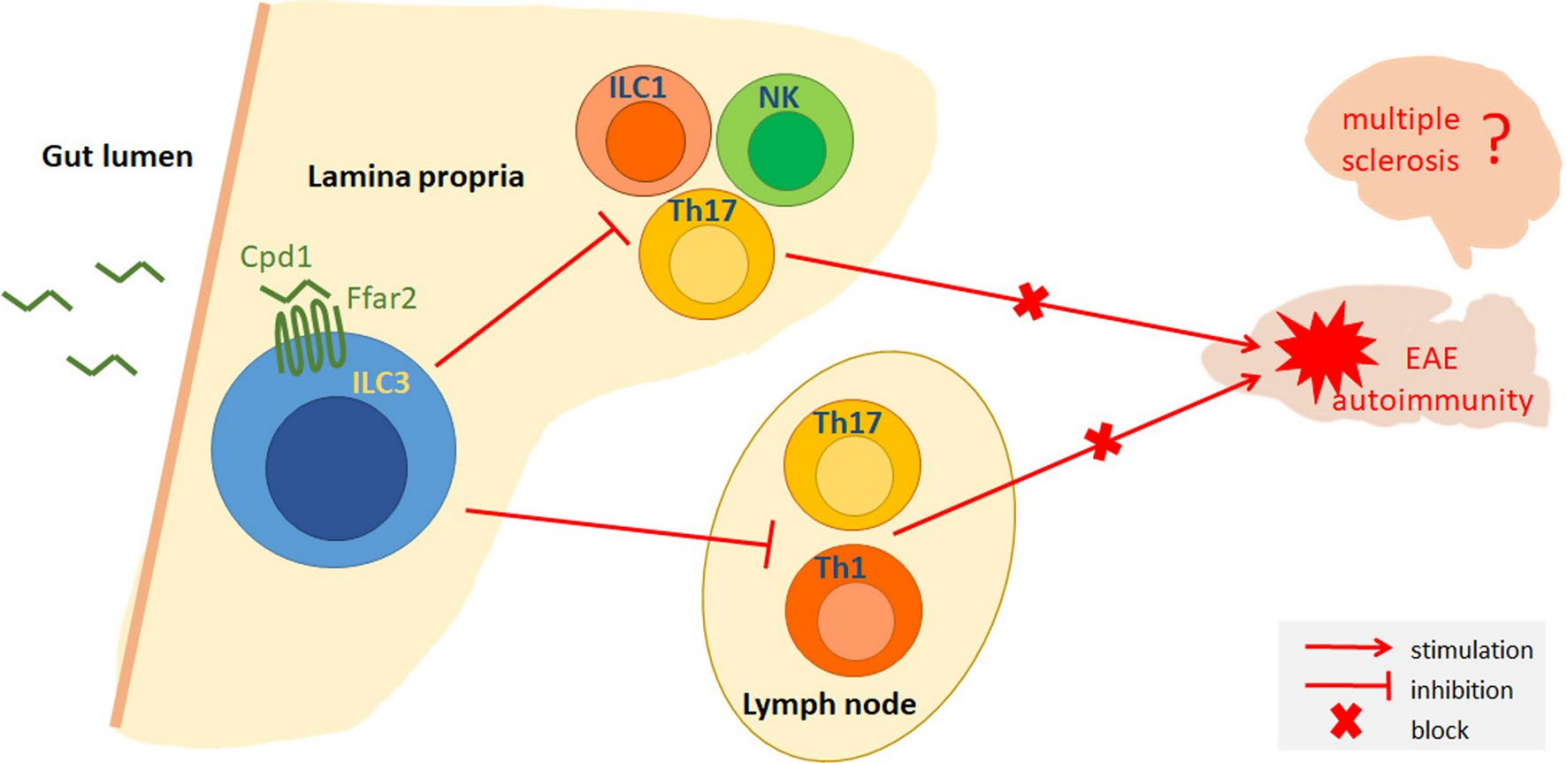
ILC3 counteracts CNS autoimmunity. ILC3 in the small intestine gut lamina propria are stimulated through Ffar2 to limit activity of encephalitogenic cells within the lamina propria, but also in other lymph organs. As the consequence of their actions CNS autoimmunity is downregulated in EAE. Future studies should reveal if such mechanism is relevant for multiple sclerosis

Different clinical outcomes are not a unique feature of SCH-induced EAE in DA rats. It is plausible to assume that divergence of clinical subgroups can be identified in each EAE variant. This study is a clear example how the imperfection of the EAE model, i.e., diverse clinical outcomes of the disease, can be used as an advantage in studying the pathogenesis of CNS autoimmunity. Specifically, our results indicate that Ffar2-mediated modulation of the immune properties of intestinal ILC3 during the induction of EAE effectively ameliorates CNS autoimmunity with clinical benefit on disease progression. Furthermore, these results imply that the gut-resident ILC3 population is a key cellular component determining clinical outcome in EAE, a commonly used model of multiple sclerosis (Fig. 10). This study highlights the importance of gut-origin ILC3 cells in CNS autoimmunity and we aim to extend these findings in further models of multiple sclerosis.

### Supplementary Information

**Additional file 1:** **Figure S1.** Th1 and Th17 gating in SCIC. **Figure S2.** CD45RA (B cell) and MHC class II gating in SCIC. **Figure S3.** Treg gating in LPIC. **Figure S4.** ILC gating in LPIC. **Figure S5.** Composition of SCIC in the moderate and severe groups. DA rats immunized with SCH were sacrificed on days 24–28 p.i. SCIC were isolated and percentage (**A**–**C**) and cell number (**D–F**) of cell populations were determined by flow cytometry. Data are expressed as mean ± SD (*n* = 5). *p < 0.05, ns—not significant. **Figure S6.** Antigen response of PLNC in moderate and severe groups. DA rats immunized with SCH were sacrificed at days 24–28 p.i. PLNC were isolated and counted (**A**). PLNC were exposed to MBP (**B**) or myelin (**C**–**G**) for 48 h. IFN-γ levels in cell culture supernatants were determined by ELISA (**B**, **C**). CD4^+^CD25^+^ T cells (**D**, **E**) and Treg (**F**, **G**) were detected by flow cytometry. Data are presented as mean ± SD (*n* = 4–5). ns—not significant. **Figure S7.** Immunoblots. Full, uncropped immunoblot images for zonulin, occludin, and actin. Samples m5, s5, m6, and s6 are presented in Fig. 6D. Blot membranes were cut in three pieces, making cuts above markers 95 kDa and 55 kDa. Protein ladder Thermo Scientific #26,619 was used. m—moderate, s—severe, h—healthy.

## Data Availability

The data sets used and/or analysed during the current study are available from the corresponding author on reasonable request.
