# Supplementary material for: Increased regulatory activity of intestinal innate lymphoid cells type 3 (ILC3) prevents experimental autoimmune encephalomyelitis severity
Source: J Neuroinflammation. 2024 Jan 18;21:26. doi: 10.1186/s12974-024-03017-7 (PMC10795263; doi:10.1186/s12974-024-03017-7)
Supplement: Supplementary file 1 — Additional file 1: Figure S1. Th1 and Th17 gating in SCIC. Figure S2. CD45RA (B cell) and MHC class II gating in SCIC. Figure S3. Treg gating in LPIC. Figure S4. ILC gating in LPIC. Figure S5. Composition of SCIC in the moderate and severe groups. DA rats immunized with SCH were sacrificed on days 24–28 p.i. SCIC were isolated and percentage (A–C) and cell number (D–F) of cell populations were determined by flow cytometry. Data are expressed as mean ± SD (n = 5). *p < 0.05, ns—not significant. Figure S6. Antigen response of PLNC in moderate and severe groups. DA rats immunized with SCH were sacrificed at days 24–28 p.i. PLNC were isolated and counted (A). PLNC were exposed to MBP (B) or myelin (C–G) for 48 h. IFN-γ levels in cell culture supernatants were determined by ELISA (B, C). CD4+CD25+ T cells (D, E) and Treg (F, G) were detected by flow cytometry. Data are presented as mean ± SD (n = 4–5). ns—not significant. Figure S7. Immunoblots. Full, uncropped immunoblot images for zonulin, occludin, and actin. Samples m5, s5, m6, and s6 are presented in Fig. 6D. Blot membranes were cut in three pieces, making cuts above markers 95 kDa and 55 kDa. Protein ladder Thermo Scientific #26,619 was used. m—moderate, s—severe, h—healthy. [file 12974_2024_3017_MOESM1_ESM.docx]

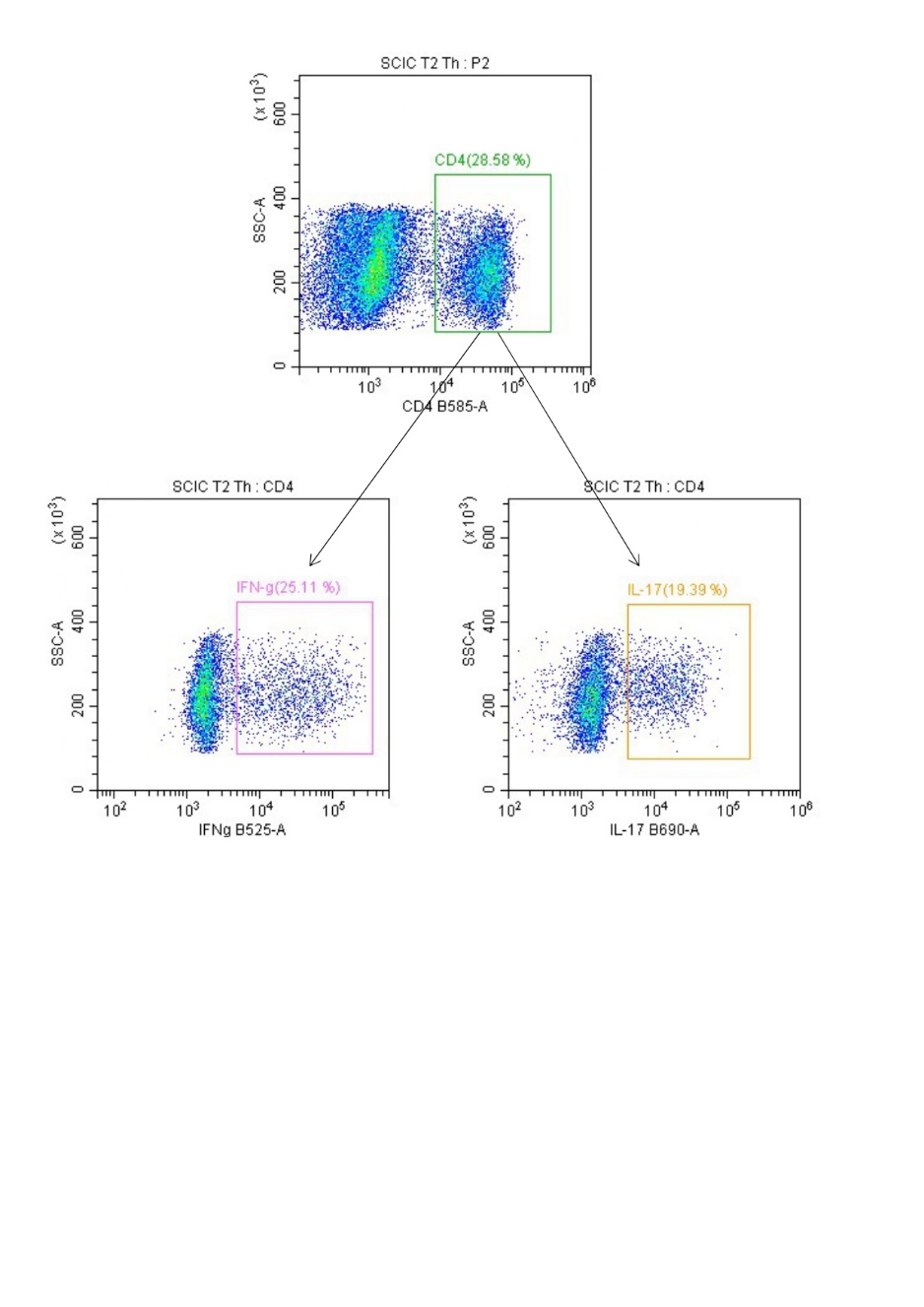


**Fig S1. Th1 and Th17 gating in SCIC.**


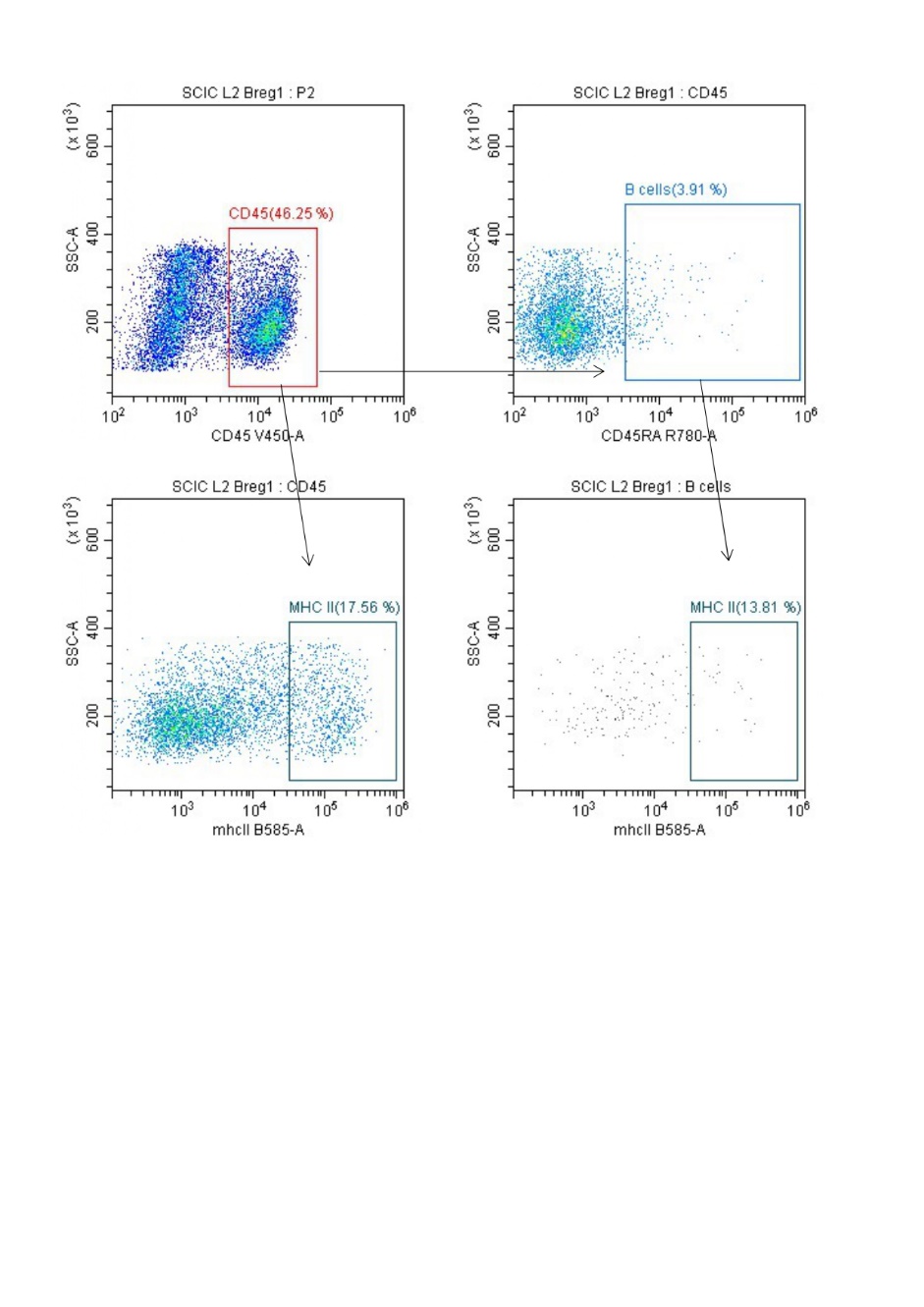


**Fig S2 CD45RA (B cell) and MHC class II gating in SCIC.**


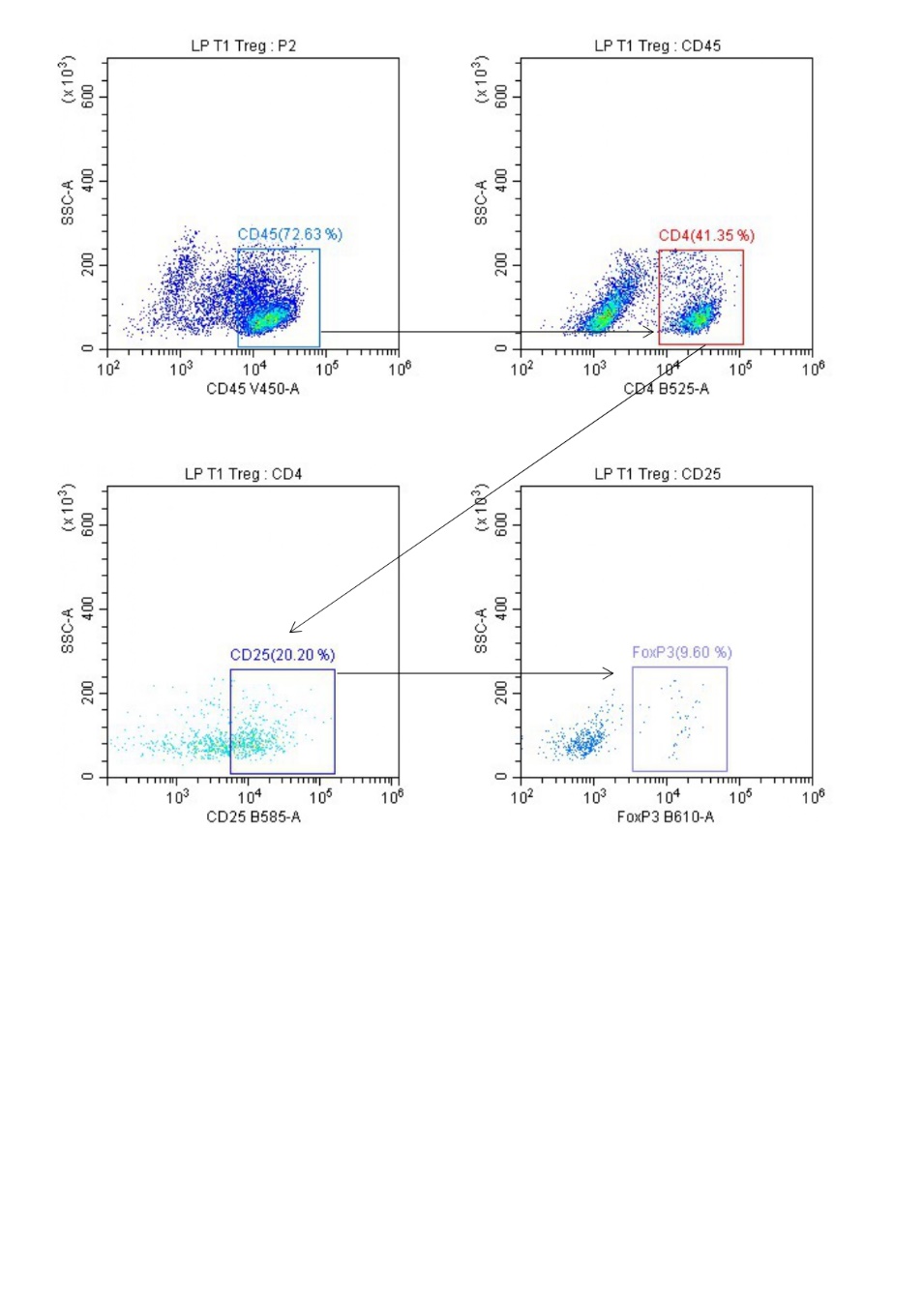


**Fig S3 Treg gating in LPIC**


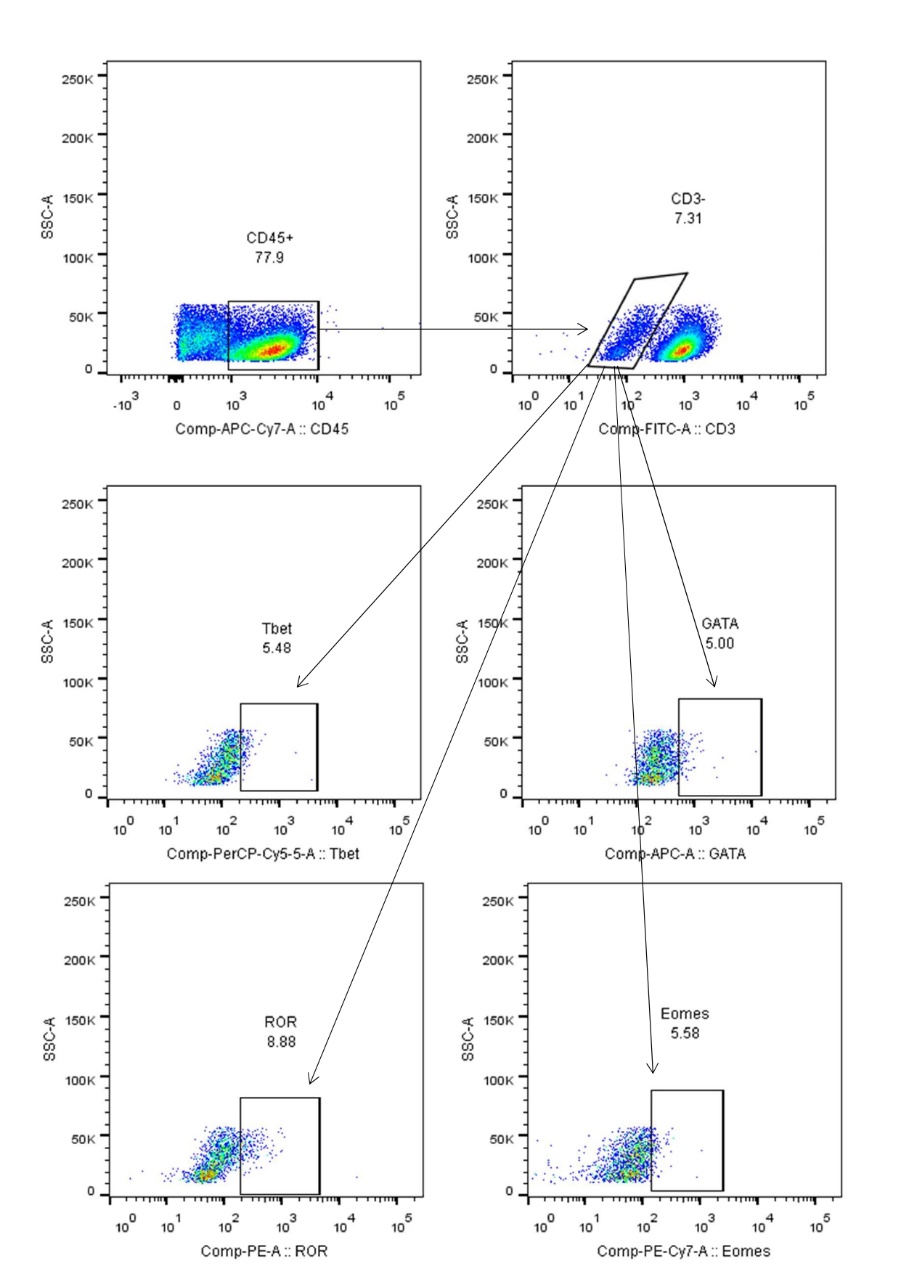


**Fig S4 ILC gating in LPIC**


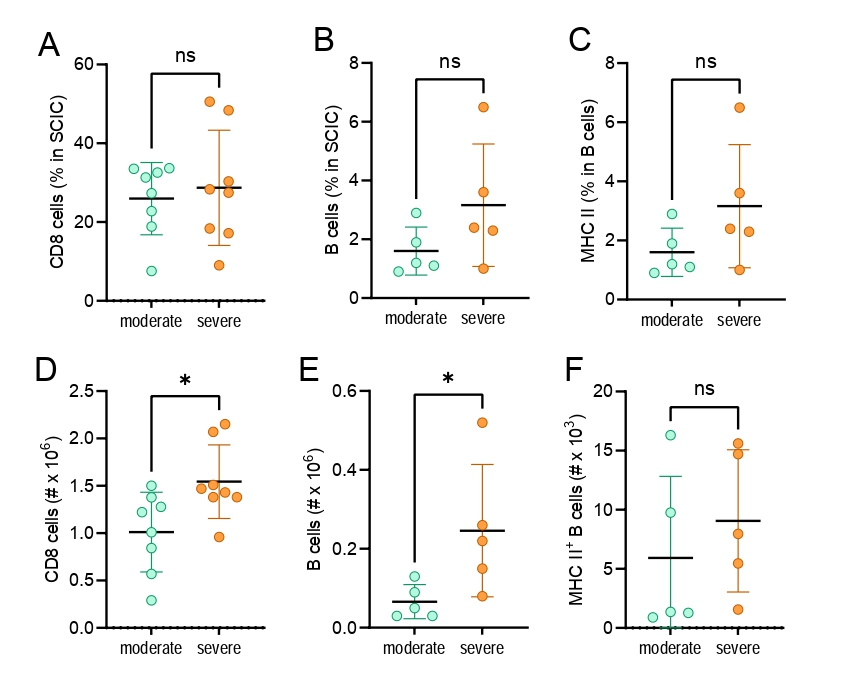


**Fig S5 Composition of SCIC in the moderate and severe groups.** DA rats immunized with SCH were sacrificed on days 24-28 p.i. SCIC were isolated and percentage (A-C) and cell number (D-F) of cell populations were determined by flow cytometry. Data are expressed as mean +/- SD (n=5). *p<0.05, ns – not significant.


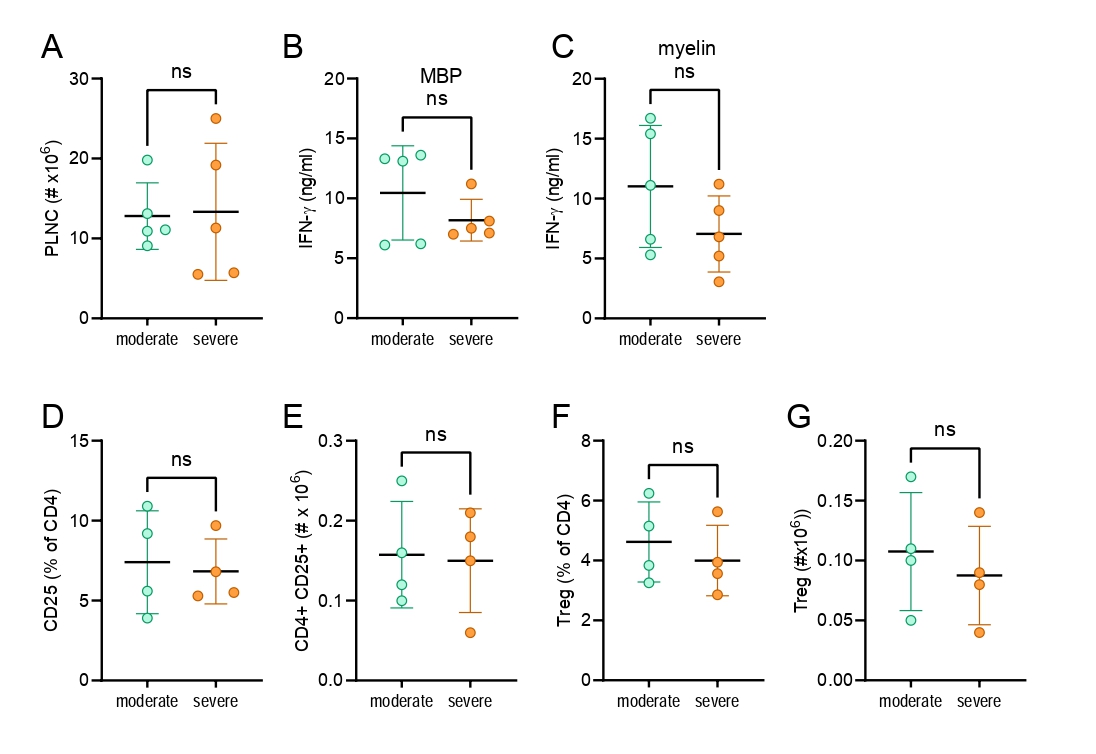


**Fig S6 Antigen response of PLNC in moderate and severe groups.** DA rats immunized with SCH were sacrificed at days 24-28 p.i. PLNC were isolated and counted (A). PLNC were exposed to MBP (B) or myelin (C-G) for 48 hours. IFN-γ levels in cell culture supernatants were determined by ELISA (B,C). CD4^+^CD25^+^ T cells (D,E) and Treg (F,G) were detected by flow cytometry. Data are presented as mean +/- SD (n=4-5). ns – not significant.


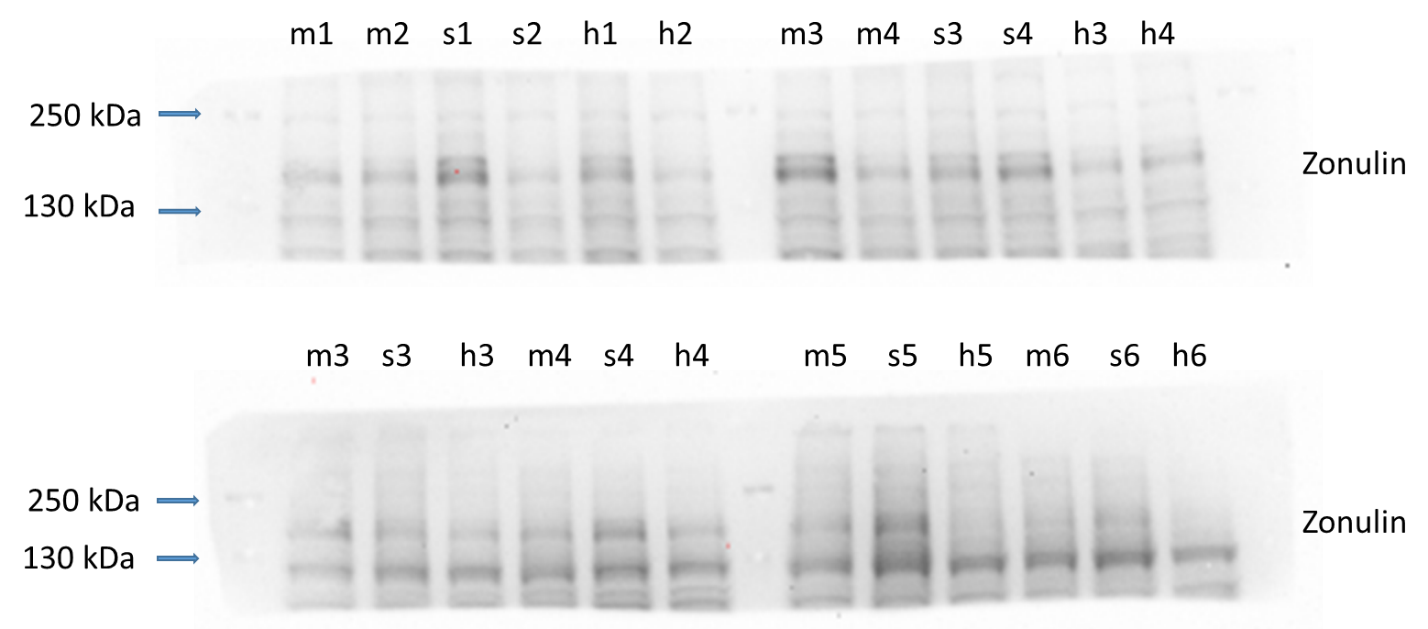


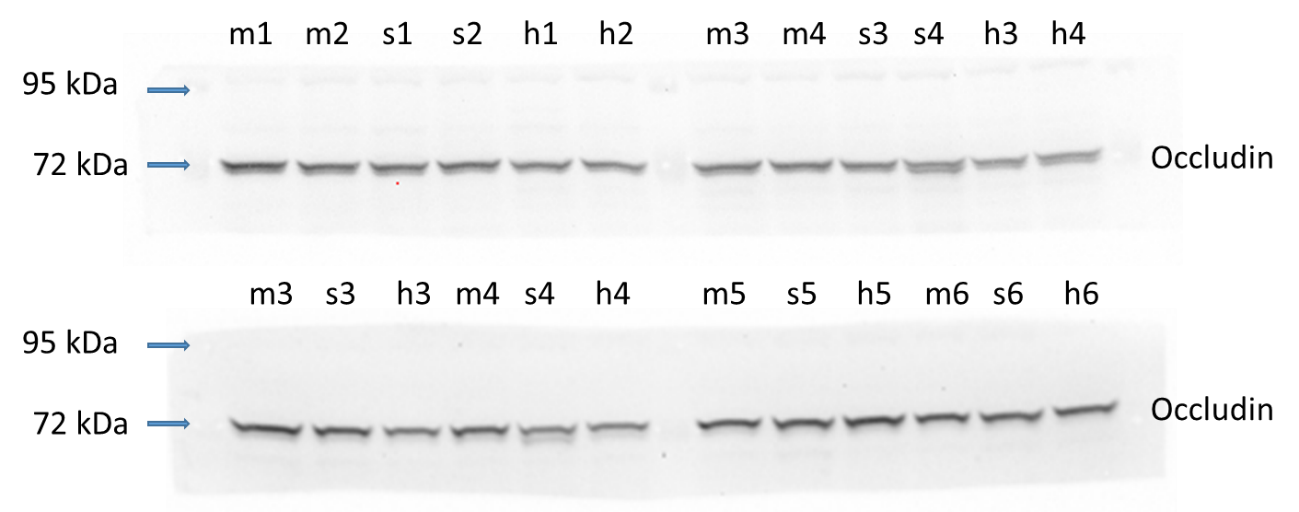


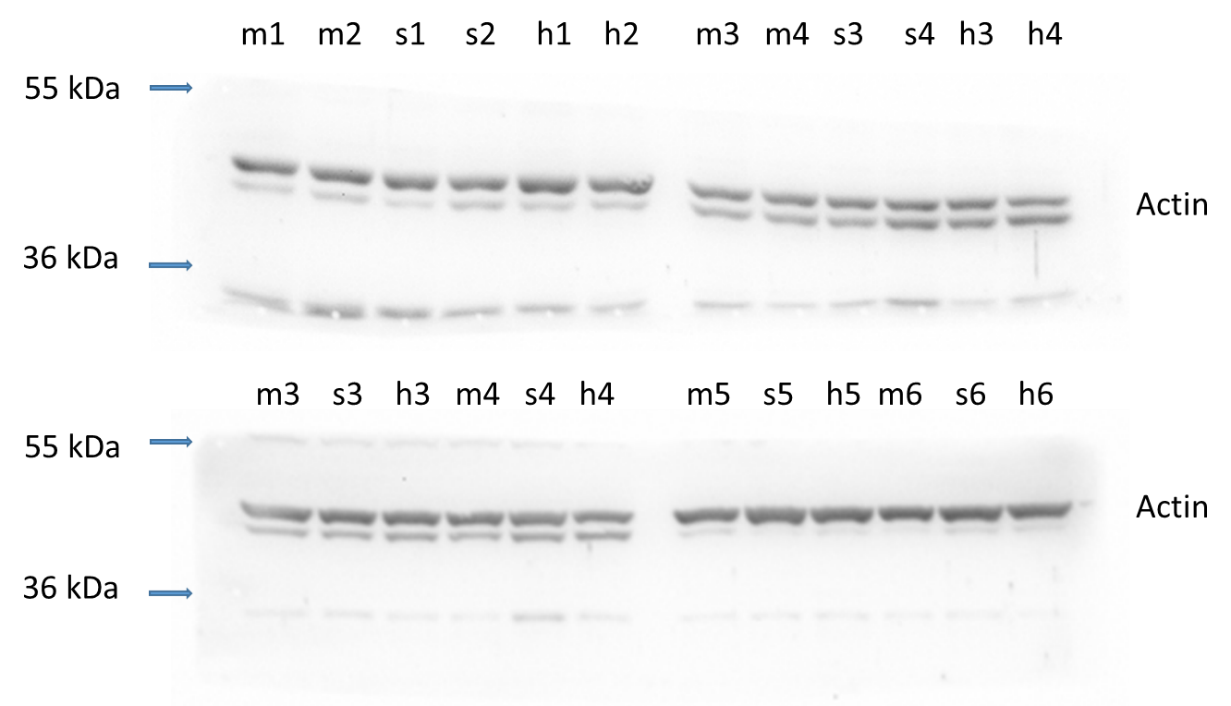


**Fig S7 Immunoblots.** Full, uncropped immunoblot images for zonulin, occludin, and actin. Samples m5, s5, m6, and s6 are presented in Fig 6D. Blot membranes were cut in three pieces, making cuts above markers 95 kDa and 55 kDa. Protein ladder Thermo Scientific #26619 was used. m - moderate, s – severe, h - healthy
